# Correlations of Genotype with Climate Parameters Suggest *Caenorhabditis elegans* Niche Adaptations

**DOI:** 10.1534/g3.116.035162

**Published:** 2016-11-17

**Authors:** Kathryn S. Evans, Yuehui Zhao, Shannon C. Brady, Lijiang Long, Patrick T. McGrath, Erik C. Andersen

**Affiliations:** * Department of Molecular Biosciences, Northwestern University, Evanston, Illinois 60208; † Interdisciplinary Biological Sciences Graduate Program, Northwestern University, Evanston, Illinois 60208; ‡ School of Biology, Georgia Institute of Technology, Atlanta, Georgia 30332; § Chemistry of Life Processes Institute, Northwestern University, Evanston, Illinois 60208; ** Northwestern Institute on Complex Systems, Northwestern University, Evanston, Illinois 60208

**Keywords:** *C. elegans*, weather, natural variation, niche, climate

## Abstract

Species inhabit a variety of environmental niches, and the adaptation to a particular niche is often controlled by genetic factors, including gene-by-environment interactions. The genes that vary in order to regulate the ability to colonize a niche are often difficult to identify, especially in the context of complex ecological systems and in experimentally uncontrolled natural environments. Quantitative genetic approaches provide an opportunity to investigate correlations between genetic factors and environmental parameters that might define a niche. Previously, we have shown how a collection of 208 whole-genome sequenced wild *Caenorhabditis elegans* can facilitate association mapping approaches. To correlate climate parameters with the variation found in this collection of wild strains, we used geographic data to exhaustively curate daily weather measurements in short-term (3 month), middle-term (one year), and long-term (three year) durations surrounding the date of strain isolation. These climate parameters were used as quantitative traits in association mapping approaches, where we identified 11 quantitative trait loci (QTL) for three climatic variables: elevation, relative humidity, and average temperature. We then narrowed the genomic interval of interest to identify gene candidates with variants potentially underlying phenotypic differences. Additionally, we performed two-strain competition assays at high and low temperatures to validate a QTL that could underlie adaptation to temperature and found suggestive evidence supporting that hypothesis.

Ecological niches describe how individuals of a species respond to, and alter the distribution of, resources and competitors within their environment (Hutchinson 1957). These resources could include food availability, soil type, short-term weather conditions, and long-term climate. Often, a species can be found in multiple distinct geographic areas that all share a common set of environmental factors and resources. For example, a plant that thrives at high temperature might grow equally well anywhere along the equator. An organism’s ability to survive in a specific niche is driven by both environmental and genetic factors. Genetic variation between species, and among individuals within a species, contributes to the wide variety of niches observed (Saltz and Nuzhdin 2014). A genetic variant could result in an increased affinity for an individual to its environment. This individual will be selected and, over time, evolution will favor the successful variant. This phenomenon, known as gene-by-environment interactions, refers to phenotypes in which different genotypes respond to environmental variation in diverse ways.

Previous studies in model organisms, particularly *Drosophila* and *Arabidopsis*, have investigated gene-by-environment interactions with clinal variation. In *Drosophila*, selection on body size is correlated with temperature (Taylor *et al.* 2015), and survival is affected by climate change (Bozinovic *et al.* 2016). Machado *et al.* (2016) performed a longitudinal study of *Drosophila* collected at differing latitudes during a two year time span and compared physiological traits of two different species: *D. melanogaster* and *D. simulans*. Other studies have gone further by identifying quantitative trait loci (QTL) for body size and cold tolerance traits involved in adaptation to seasonally varying environments (Tyukmaeva *et al.* 2015; Hangartner *et al.* 2015). Gerken *et al.* (2015) found substantial heritable variation in both short-term and long-term acclimation. They then performed genome-wide association (GWA) mappings on these traits and found the QTL for short-term and long-term adaptations did not overlap, but each resulted in a set of gene candidates sharing similar functions in apoptosis, autophagy, cytoskeletal and membrane structural components, or ion binding and transport. Additionally, GWA studies have been performed in *Arabidopsis* for various adaptive traits such as seed oil melting point (Branham *et al.* 2016). Another *A. thaliana* study performed QTL mapping using recombinant inbred lines produced from two strains isolated from different climates and found that relatively few QTL explain much of the adaptive divergence between them (Ågrena *et al.* 2013). Furthermore, Fournier-Level *et al.* (2011) provide evidence for broad scale local adaptation in *A. thaliana* by using a GWA mapping approach that combines fitness traits measured in multiple natural environments, and geographic and climatic analyses.

*Caenorhabditis elegans* is a free-living nematode often found in microorganism-rich organic material such as rotting fruits and compost heaps in temperate and humid environments (Frézal and Félix 2015; Félix and Braendle 2010). The first studied *C. elegans* strain, N2, was isolated from mushroom farm compost in Bristol, England in 1951 (Hodgkin and Doniach 1997). Since that time, N2 has been used as the wild-type strain for *C. elegans* laboratory research. This strain was cultured for many years in the laboratory, potentially resulting in selection for alleles favorable in that environment (Sterken *et al.* 2015). To study natural variation and the role of niche specification on this species, we require a worldwide collection of wild strains. Our research group acquired a large collection of 208 wild strains and sequenced the whole genomes of these strains (Cook *et al.* 2016a,b; Thompson *et al.* 2015). By comparing the genomes of the 208 strains, we found that some strains from similar geographic locations have nearly identical genome sequences. This analysis resulted in 152 unique genome-wide haplotypes or isotypes. This large pool of genetic information provides us with the statistical power to make connections between genotype and phenotype using GWA studies.

A handful of studies have addressed differences in temperature sensitivities across *C. elegans* strains, and many of these studies show that temperature affects the lifetime fecundity and reproductive timing of *C. elegans*. Two separate groups used a small subset of wild isolates to assay thermal tolerance (Petrella 2014), and thermal sensitivities for fitness traits (Anderson *et al.* 2011). Other studies performed linkage mappings using a recombinant inbred line panel of *C. elegans* strains to map life history traits such as fertility, growth rate, and egg size, at both low and high temperatures to various locations across the genome (Gutteling *et al.* 2007a,b). However, the use of population-scale *C. elegans* data to map differences found in a large number of wild isolates for natural environmental conditions has yet to be addressed. In this study, we correlate natural genetic variation among 152 wild *C. elegans* strains with climate measurements of their environmental niches as quantitative traits. We mapped traits that describe the niche of the isolation location for each strain, including geographic parameters, seasonal weather patterns, and climate variables. We find significant associations for elevation, relative humidity, and temperature. These findings suggest genetic control of niche specification. Additionally, we tested the QTL associated with temperature and found possible evidence of adaptation to lower temperatures based on genetic background.

## Materials and Methods

### C. elegans wild isolate collection and sequencing

A collection of 208 wild *C. elegans* strains has been previously isolated worldwide and annotated for each strain’s geographic location and date of isolation (Cook *et al.* 2016b). Members of the Andersen laboratory have carefully and manually curated this dataset to offer the most accurate information possible while accounting for sometimes imprecise sample recording. Whole-genome sequence (WGS) data were collected from all 208 strains (Cook *et al.* 2016b). The raw Illumina data are deposited with the Short Read Archive under project PRJNA318647. WGS data were analyzed as previously described (Cook *et al.* 2016b). In brief, after alignment with *Burrows-Wheeler Alignment* (Li and Durbin 2009) and variant calling using Samtools (Li *et al.* 2009), strains with a concordance of 99.93% or higher were grouped as a genome-wide haplotype or isotype. This analysis resulted in 152 unique isotypes (Supplemental Material, File S1).

### Weather and climate data acquisition

For each wild strain with a known isolation location, elevation was estimated with the “geosphere” package in *R* (Hijmans *et al.* 2012), using the geographic coordinates of strain isolation. A correlation test using 74 points of known elevation was used to verify accuracy of the elevation function, resulting in a correlation of 0.998. Weather data were downloaded from the Integrated Surface Data (ISD) FTP server (ftp://ftp.ncdc.noaa.gov/pub/data/noaa/) managed by the National Oceanic and Atmospheric Administration (NOAA), and the National Climatic Data Center (NCDC). The ISD dataset comprises worldwide surface weather observations from over 27,447 stations managed by the Automated Weather Network (AWN), the Global Telecommunications System (GTS), the Automated Surface Observing System (ASOS), and others. Data are collected once every 3 hour for some stations. Some parameters include air quality, atmospheric pressure, atmospheric temperature, dew point, atmospheric winds, clouds, precipitation, ocean waves, and tides.

Three distinct sets of weather station data were collected for analysis: a 3-month window, a one-year window, and a three-year window. These data were filtered to include values centered around the date of isolation. For the 3-month set, wild isolates with only a known month or year of isolation were not considered. Exact day of isolation is necessary to understand the seasonal environment in which an animal was isolated. For the one-year dataset, wild isolates with a month or day of isolation were used. For the three-year dataset, strains with only a year of isolation were used in addition to those strains with more defined dates of isolation. If only the year was known, the date of isolation defaulted to January 1 of that year, and data were collected surrounding that date. If only the month of isolation was known, the date of isolation was defaulted to the first of that month for data collection.

The 27,447 NOAA weather stations were filtered by their availability of data collected within the years of interest. Stations that had fewer than 10 recordings of any type for any month within the time period of data collection were excluded to avoid misrepresentation by datasets that were averaged from only a few data points. Stations were then filtered by location, and the closest station to location of isolation for each wild strain was selected and downloaded using the “stationaRy” package available at https://github.com/rich-iannone/stationaRy (Iannone 2015). We performed a rank-correlation test for temperature, relative humidity, and atmospheric pressure between two neighboring weather stations (ranging from 0.93 to 153 km apart) and found high correlations regardless of distance between stations (*rho* = 0.920, 0.913, and 0.707, respectively). All station-isotype pairs were included in our analysis, regardless of the distance between them. The primary fields, as well as all additional quantitative data available for each station, were downloaded. Some fields (*e.g.*, “AT1” or “Present-weather-observation”) were not downloaded from the NOAA database because the traits are qualitative and would not be conducive to quantitative analyses. The station data were filtered to contain only information from the months surrounding the date of isolation. This process was repeated for each dataset (3-month, one-year, and three-year) in case a closer weather station contained only data for the 3-month set, but not for the one- or three-year sets. The station data were meticulously checked by manually removing missing values from each weather category independently and, in certain cases, converting fields to uniform units that can be averaged to form a trait value. For example, precipitation (“AA1”) was downloaded in two columns: (1) time period; (2) depth of precipitation recorded during that time period. Because the variable time periods in which data were recorded, averaging precipitation would lead to skewed results. Precipitation was changed to adapt a “precipitation per hour” model that would be more permissive to our analyses. The daily average of each trait was averaged over the time span collected (3 months, one year, or three year), and this value was designated as the phenotypic value for that strain. Furthermore, the minimum and maximum daily values and total variance were averaged over the time span collected. Only traits with values in greater than 90% of strains were analyzed further.

### Association mapping

GWA mapping was performed using 152 genome-wide *C. elegans* isotypes with the cegwas *R* package found at https://github.com/AndersenLab/cegwas. This package uses the EMMA algorithm for performing association mapping and correcting for population structure (Kang *et al.* 2008), which is implemented by the GWAS function in the rrBLUP package (Endelman 2011). The kinship matrix used for association mapping was generated using a whole-genome high-quality single nucleotide variant (SNV) set (Cook *et al.* 2016b), and the A.mat function from the rrBLUP package. Single-nucleotide variants identified using RAD-marker sequencing (Andersen *et al.* 2012) that had at least 5% minor allele frequency in the 152 isotype set were used for performing GWA mappings. Association mappings that contained at least one SNV that had a −log(*p*-value) greater than the Bonferroni-corrected *p*-value were processed further using fine mapping, which entails a Spearman’s rank correlation test with variants from the WGS data of moderate-to-severe predicted effects as determined by the SnpEff function (Cingolani *et al.* 2012).

### Temperature competition assays

We chose two strains, CX11314 and JU847, that had different alleles for the peak QTL marker (chrV: 14,822,276; JU847: T, CX11314: A) in our three-year temperature GWA mapping. JU847 has the reference allele for the peak marker, and was isolated at a low temperature, whereas CX11314 has the alternative allele for the peak marker and was isolated at a higher temperature. We designed a Taqman probe (5′-[A]CCGTTTTTTTT[T/A]AATTTT-3′) to measure each of these two alleles from mixed samples of nematodes using the standard software from Applied Biosystems (https://www.thermofisher.com/order/custom-genomic-products/tools/genotyping/) and a corresponding primer set to amplify the region of interest (below).

F: 5′-AAACCCAAGATTTTTATGGTTACTTTAAGATTTGT-3′;
R: 5′-ATCTATAGTTAACTTGGATATATTGTTTGTTTTCGGT-3′

These two strains were chunked to fresh 10 cm NGMA plates seeded with OP50; 48 hr later, seven L4s from each strain were added to each of 45 6-cm NGMA plates seeded with OP50 for both 15° and 25° competition experiments. The 45 plates at each temperature represent nine experimental replicates, each composed of five independent populations. Plates were placed at either 15 or 25°, and grown to starvation. After one week for 25° competition experiments, or 10 d for 15° competition experiments, nematodes were transferred to fresh NGMA plates by cutting a 0.5 × 0.5 cm square of agar (containing ∼100 worms) and replaced at the appropriate temperature. After culture transfers, two, four, and six, starved animals were washed off the plates with M9, and DNA was collected using the Qiagen DNeasy Kit. Genomic DNA from each time point was digested with the *Eco*RI enzyme and purified using the Zymo DNA Clean and Concentrator Kit. The concentration of fragmented genomic DNA was adjusted to 2 ng/µl by Qubit assay. The number of JU847 and CX11314 alleles in each replicate population was measured using Taqman analysis in a Bio-Rad QZ200 digital droplet PCR system (File S4). Digital PCR was performed following the standard protocol provided by Bio-Rad with the absolute quantification method. The proportion of the JU847 allele and the relative selection coefficients were calculated.

### Data availability

Strains are available through the *C. elegans* Natural Diversity Resource (CeNDR, www.elegansvariation.org). File S1 contains the strains, location data, weather station data, and all traits used in mappings. File S2 contains the association mapping data. File S3 contains the interval fine mapping raw data. File S4 contains the count data from the digital droplet PCR for JU847 and CX11314 alleles.

## Results

### GWA of geographic traits

**Figure 1:**
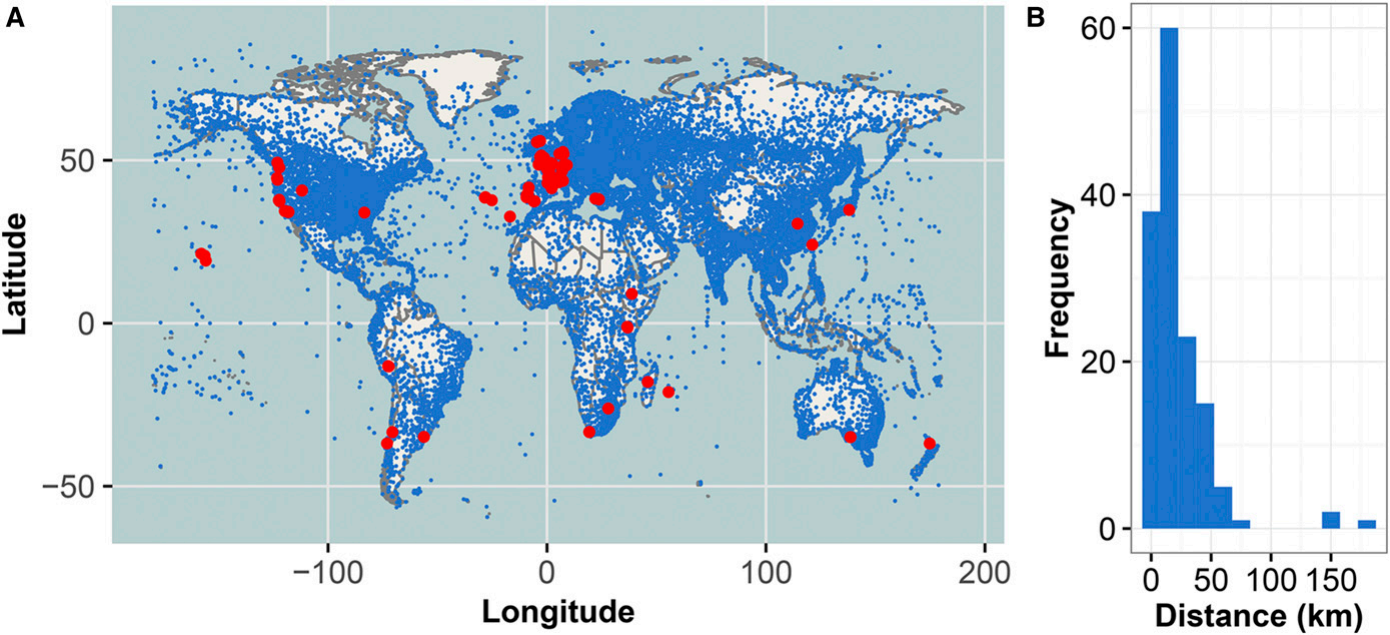
Global distribution of wild isolates and NOAA weather stations. (A) Map of 27,447 ISD NOAA weather stations (blue), and 149 *C. elegans* wild isolate locations (red). Three isotypes are not depicted as they have no known location of isolation. (B) Histogram of station distance from wild isolate, measured in km. Data from the three-year weather dataset are shown.

**Figure 2:**
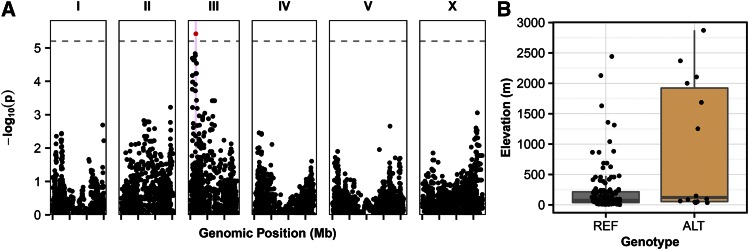
GWA of elevation. (A) GWA of elevation of strain isolation shown as Manhattan plots. Genomic position is plotted on the *x*-axis against the negative log-transformed *p*-value on the *y*-axis. SNVs that are above the Bonferroni-corrected significance threshold, indicated by the dotted gray line, are shown in red, and SNVs below the Bonferroni threshold are shown in black. Confidence intervals are represented by the pink bars. (B) Box plots show strain isolation elevations (m), separated by the genotype at the peak marker location. Each point represents one strain. The reference genotype (REF) refers to strains that share the genotype of the reference strain, N2. The alternative genotype (ALT) refers to all other strains that do not have the reference genotype at the peak marker position.

The location where a *C. elegans* strain was identified could reflect the process of selection for a particular genotype in a specific niche. For this reason, we investigated correlations between genetic variation in the *C. elegans* population and parameters describing the geographic locations of isolation as quantitative traits. Previous work with a smaller set of strains (97 wild isolates) detected a significant QTL on the left arm of chromosome II associated with the latitude where strains were isolated (Andersen *et al.* 2012). To evaluate this trait and other parameters describing the location where each strain was isolated in our larger strain set (152 strains), we curated the isolation location information, and defined several traits based on geographic data for each strain (Table 1), namely latitude, longitude, elevation, the absolute value of latitude, and the absolute value of longitude (File S1). We performed GWA mappings with 149 wild strains with known isolation locations (Figure 1A) to correlate these trait values with common genetic variation (File S2; see *Materials and Methods*). Using this strain set, only the mapping of the elevation of the isolation location identified a significant QTL on the left arm of chromosome III (Figure 2A). When we divided the population by the genotype at the peak marker, we found that the elevation values for these two sets of strains were similar with a few outliers (Figure 2B), suggesting that the outliers were causing the detection of a QTL. It is possible that the association is spurious and driven by outliers, or that the outliers are strains harboring rare alleles in the *C. elegans* species that impact this trait. The outlier strains in our mapping could share some genetic similarities as they were all collected within the last 15 years, and most were collected in France or elsewhere in Northern Europe. We did not recapitulate the QTL for latitude observed in the previous study (Andersen *et al.* 2012), likely because it also appears to be driven by strains with extreme latitude values. Again, these outlier strains could be highly related, as seven of the 15 strains with the alternate genotype at the peak marker position originated from South Africa or Kenya. Our larger strain set reduces the effect of these outliers on the GWA mapping, and the previously detected QTL is no longer significant. These results suggest that common variation in the *C. elegans* species does not correlate with geographic parameters describing the location of strain isolation. However, rare variants might control whether strains can colonize, and/or proliferate, in specific geographic locations.

**Table 1:** Definition of geographic and weather traits

Trait Name	Trait Code*^a^*	Number of Strains*^b^*	Description
Latitude	latitude	149	Latitude coordinate where the nematode strain was collected (°)
Absolute value of latitude	abslat	149	Absolute value of the latitude coordinate where the nematode strain was collected (°)
Longitude	longitude	149	Longitude coordinate where the nematode strain was collected (degrees)
Absolute value of longitude	abslong	149	Absolute value of the longitude coordinate where the nematode strain was collected (°)
Elevation	elevation	149	Calculated elevation based on latitude/longitude coordinates using “geosphere” package (m)
Temperature	temp	145	Average daily*^c^* temperature of the air (°)
Relative humidity	rh	129	Amount of water vapor present in air expressed as a percentage of the amount needed for saturation at the same temperature (%)
Wind direction	wd	134	Angle, measured in a clockwise direction, between true north and the direction from which the wind is blowing (°)
Wind speed	ws	136	Rate of horizontal travel of air past a fixed point (m/sec)
Cloud height*^d^*	ceil_hgt	122	Height above the ground level of the lowest cloud or obscuring phenomena layer with 5/8 or more summation total sky cover, which may be predominantly opaque, or the vertical visibility into a surface-based obstruction (m)
Dew point	dew_point	129	Temperature to which a given parcel of air must be cooled at constant pressure and water vapor content in order for saturation to occur (°)

Definitions for traits obtained from the Federal Climate Complex Data Documentation for Integrated Surface Data http://www1.ncdc.noaa.gov/pub/data/ish/ish-format-document.pdf (August 20, 2015).

a Abbreviation for weather traits obtained from the raw data file format.

b Number of wild isolates with data for each geographic and weather trait. Number of strains for the weather traits was obtained from the 3-month mapping dataset.

c All weather traits were defined by averaging the average daily values (see *Materials and Methods*).

d Cloud height data were only sufficient to analyze for the three-year dataset.

### Weather conditions and climate parameters can be determined using the geographic location of the site of strain isolation

It is likely that the possible genetic association we observed between the elevation of strain isolation and a region on chromosome III is correlated with weather patterns and/or climate variables at specific geographic locations. Strain latitude and longitude can be used to determine the weather or climate at the time and location from which each wild *C. elegans* strain was isolated. The short-term weather surrounding the day of isolation, as well as the long-term climate of the geographic location for each strain could improve our understanding of adaptation to niches for specific strains or for the species as a whole. Furthermore, correlating these climate parameters with whole-genome sequence data could identify potential alleles that might contribute to adaptation of a wild isolate to certain environmental factors.

NOAA collects and provides multiple datasets related to weather and climate information, including the ISD. The ISD dataset is archived at the NCDC and is composed of worldwide surface weather observations from over 27,000 stations managed by different global institutions (NOAA Data Catalog 2016). First, we manually curated the isolation information for the 152 strains, including the date of isolation, location, and sampling information (File S1). Then, we overlaid the locations of the 27,447 ISD weather stations with the isolation locations of the 149 *C. elegans* wild strains with complete sampling data (Figure 1A). Using the date and isolation location for each wild strain, we identified the closest weather station with available data and collected weather observations in three time periods surrounding the date of nematode isolation: 3 months, one year, and three year. Most strains were found <60 km from a weather station (Figure 1B). However, we found a strong correlation (*rho* = 0.707) between weather stations up to 150 km apart, suggesting the weather station assigned to each strain is representative of the weather and climate from which the strain was isolated. Of the 149 strains with known isolation locations, we knew at least the year of isolation for 145 strains, the month for 138 strains, and the day for 122 strains. For the 3-month period, we analyzed weather station data only for strains with known days of isolation to provide a precise account of the daily weather experienced immediately surrounding the date of isolation for each strain. For the one-year period, we used data from strains with known day or month of isolation. For the three-year period, we used data from strains with a known day, month, or year of isolation to provide an estimated overall climate of the strain isolation location. Not every weather station sampled contained data for each weather parameter. Additionally, only quantitative weather parameters that were measured at locations shared in a majority of the wild isolate population (>90% of the strains) were considered for further analysis (Table 1). The daily averages of all observations for each weather parameter were averaged over the given time period, and this averaged value was used as the trait measurement for each strain (File S2, see *Materials and Methods*).

We evaluated all weather observations over the 3 months, one year, or three year surrounding the date of nematode isolation for each of the 149 wild strains to define the weather or climate experienced by each strain. These data were mapped using GWA to define 10 QTL (Figure S1) for two distinct traits: relative humidity and temperature.

### GWA of average relative humidity

**Figure 3:**
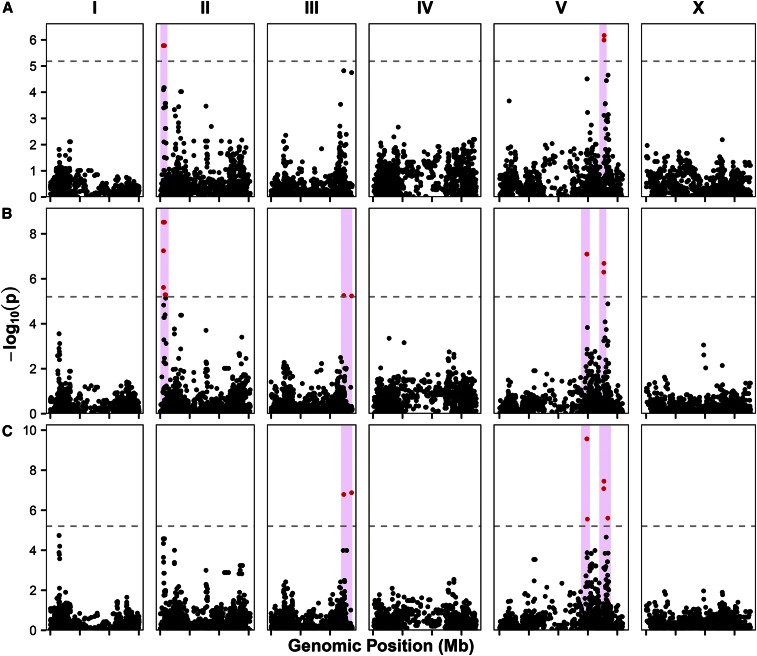
GWA of humidity traits. GWA of relative humidity for three different time periods are visualized as Manhattan plots: 3-month (A), one year (B), three year (C) durations. Genomic position is plotted on the *x*-axis against the negative log-transformed *p*-value on the *y*-axis. SNVs that are above the Bonferroni-corrected significance threshold, indicated by the dotted gray line, are shown in red, and SNVs below the Bonferroni threshold are shown in black. Confidence intervals are represented by the pink bars.

*C. elegans* is found at locations with various average daily relative humidity, ranging from 34 to 89%, with an average of 70% (File S2). This estimate of the average relative humidity of locations harboring *C. elegans* is in agreement with previous studies that show *C. elegans* is often found in humid environments (Frézal and Félix 2015). To determine if variation in relative humidity at isolation location is correlated with genetic variation, we performed GWA mapping for relative humidity of isolation location over 3 months, one year, and three year surrounding the date of isolation (see *Materials and Methods*). We found nine significant QTL in three distinct regions of the *C. elegans* genome: the left arm of chromosome II, the right arm of chromosome III, and the right arm of chromosome V (Figure 3 and File S2). The mapping for average relative humidity over 3 months surrounding the date of isolation (Figure 3A) resulted in two distinct QTL: one on the left arm of chromosome II and the other the right arm of chromosome V (LD *r*^2^ = 0.341; Figure S1). The mapping for average relative humidity over one year surrounding the date of isolation (Figure 3B) resulted in one QTL on the left arm of chromosome II, two linked QTL on the right arm of chromosome V (LD *r*^2^ = 0.536; Figure S1), and one barely significant QTL on the right arm of chromosome III that is highly linked to the other three QTL (LD *r*^2^ = 0.774, 0.777, 0.548; Figure S1). The position of the chromosome II and V QTL are the same as those QTL observed for the mapping of 3-month humidity. Finally, the mapping of average relative humidity over the three year surrounding the date of isolation (Figure 3C) resulted in the same two QTL on chromosome V and the QTL on chromosome III, as found for the mapping of one-year relative humidity. For each QTL, strains with the reference allele at the peak marker tend to be isolated at higher relative humidity, and strains with an alternative allele tend to be isolated at lower relative humidity (Figure S1). This evidence of a phenotypic split dependent on genotype of the peak marker suggests that at least one variant could contribute to the adaptation of *C. elegans* to different relative humidity.

### GWA of average temperature

**Figure 4:**
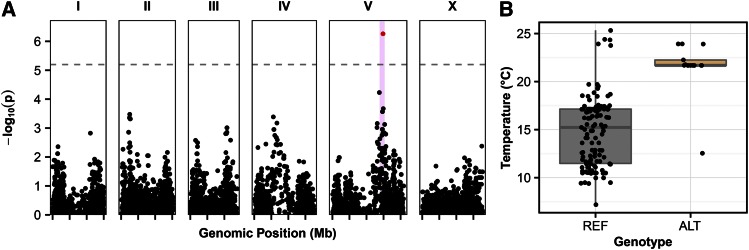
GWA of temperature. (A) GWA of three-year average temperature is visualized as a Manhattan plot. Genomic position is plotted on the *x*-axis against the negative log-transformed *p*-value on the *y*-axis. SNVs that are above the Bonferroni-corrected significance threshold, indicated by the dotted gray line, are shown in red, and SNVs below the Bonferroni threshold are shown in black. Confidence intervals are represented by the pink bars. (B) Box plots show the strain three-year average temperatures, measured in degrees Celsius, separated by genotype at the peak marker locus. Each point represents one strain. The reference genotype (REF) refers to strains that share the genotype of the reference strain, N2. The alternative genotype (ALT) refers to all other strains that do not have the reference genotype at the peak marker position.

*C. elegans* are also found at a variety of average temperatures, ranging from 7 to 25°, with an average of 15.3° (File S1). To determine if temperature is associated with genetic variation, we performed a GWA mapping for the average daily temperature of isolation location over 3 months, one year, and three year surrounding the date of isolation (see *Materials and Methods*). We found one significant QTL just right of the center of chromosome V (Figure 4A and File S2). This QTL is in the same location as that observed for relative humidity (Figure 3). We found that strains with the reference (N2) allele at the peak marker tend to be isolated from geographic locations with lower temperatures, and strains with an alternative allele at this position tend to be isolated from geographic locations with higher temperatures (Figure 4B). This QTL suggests that an allele or alleles nearby this marker could confer fitness advantages to strains that experience different temperatures. To identify the variant(s) that could underlie this QTL, we investigated a region on chromosome V (V:13,845,281–15,332,878) defined by 1.48 Mb that contains 619 total genes. The genes with predicted functional variants are most likely to cause phenotypic differences among diverse strains in species. Therefore, we focused on 363 genes within this region predicted to harbor functional variants of moderate or severe effects on gene function, as determined by SnpEff (Cingolani *et al.* 2012). We narrowed the list of candidate genes further by identifying 27 genes that are highly correlated with differences in temperature (File S3). Although an investigation of these 27 genes did not identify an obvious candidate related to temperature regulation, one or more of these variant genes could explain adaptation to specific temperatures.

Because *C. elegans* are isolated at locations with variable temperatures, we wanted to investigate the variance of temperatures experienced by each strain. We found one significant QTL associated with the variance in annual temperature on chromosome V and another on the right arm of chromosome III (Figure S1). These QTL appear to be linked (LD *r*^2^ = 0.661) but act in opposing manners. On chromosome III, strains with the reference allele at the peak marker tend to experience lower variance in annual temperature than strains with the alternative allele at this position. However, the strains with the reference allele at the peak marker for the QTL on chromosome V tend to experience higher variance in annual temperature than those strains with the alternative allele (Figure S1). Furthermore, the average minimum daily temperature for three year experienced by each strain also maps to the same chromosome V QTL, while the variance in temperature over the three-year window maps to the same QTL previously identified on chromosome III (Figure S1). Although the same strains have both the alternative allele at the peak marker for average temperature and minimum temperature (and are mostly found in either Southern California or Hawaii), only a few of these strains also have the alternative allele for variance in temperature. Taken together, these data are suggestive of genotypic differences that exist within the strains harboring the alternative allele that highly correlate with being found in locations with higher temperatures and lower temperature variation.

### Strains from divergent climates might be adapted to specific temperatures

Although we have GWA mappings for various weather conditions, validating these QTL would provide more evidence for *C. elegans* selection of niche based on environmental and geographic factors. Because temperature can be controlled easily, and survival at defined temperatures can be tested experimentally, we decided to determine whether two strains from divergent climates are adapted to the respective temperatures nearby their isolation locations using a competition assay. Strains were chosen that had both a different genotype at the peak marker of the QTL on chromosome V identified in the three-year temperature mapping experiment and a large difference in the temperatures nearby the isolation locations. JU847, a strain isolated from Northern France in 2005 (File S1), has the reference genotype at the three-year temperature QTL peak marker and was found at a low three-year average temperature (11.3°). CX11314, a strain isolated from Southern California in 2003 (File S1), has the alternative genotype at the three-year temperature QTL peak marker and was found at a higher three-year average temperature (20.9°). High fitness at or around the temperature nearby the isolation location of one strain and lower fitness at or around the temperature nearby the isolation location for the other strain would suggest potential adaptive alleles that contribute to better survival at a specific temperature.

**Figure 5:**
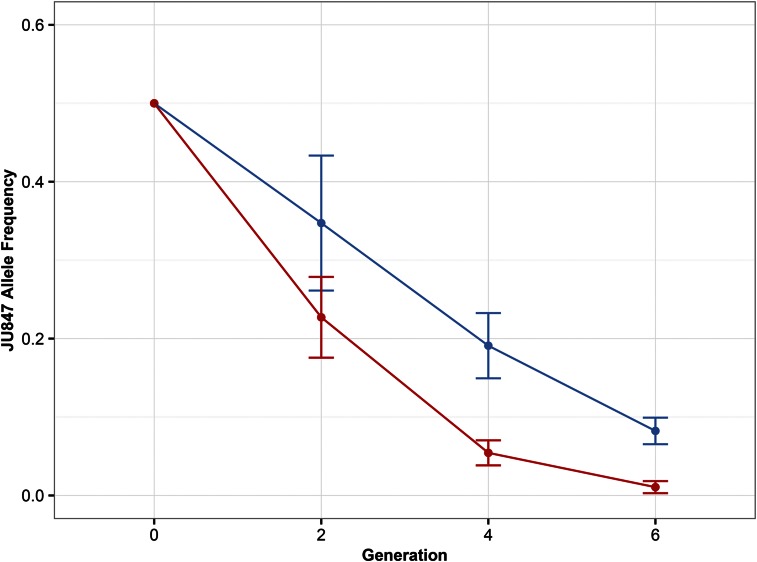
Two-strain temperature competition assay. JU847 (isolated at low temperature) was competed against CX11314 (isolated at high temperature) at both 15° (indicated in blue) and 25° (indicated in red). The mean frequency of the JU847 allele in the population is plotted on the *y*-axis. Error bars represent 1 SD from the mean. Data were collected from nine experimental and five technical replicates.

Replicate cultures were initiated with an equal number of animals from each strain at either 15 or 25° and allowed to compete for at least six generations. After culture transfers two, four, and six, we analyzed the ratios of the two strains found at each temperature (see *Materials and Methods*). CX11314 was found to have higher fitness than JU847 at both temperatures tested (Figure 5). At high temperature, CX11314 had a clear selective advantage compared to JU847 (for fitness = 1, relative CX11314 fitness *s* = 2.29), resulting in JU847 alleles comprising fewer than 1% of the alleles measured after six culture transfers. However, JU847 performed better at the lower temperature than at the higher temperature, comprising almost 8% of the total nematode population after six culture transfers (relative CX11314 fitness *s* = 1.57). These data suggest that JU847, although not more fit than CX11314 at either temperature, is more fit at 15° than at 25°.

## Discussion

In this study, we have defined geographic, weather, and climate variables over three different time periods as phenotypic traits for 149 unique wild *C. elegans* strains and performed GWA mapping for 11 traits. Each phenotype described in this study was obtained using the location, date, and weather of the known isolation location of each isotype in our collection. We found significant correlations between genotype and phenotype for three traits and a total of 11 QTL. However, only temperature displayed strong phenotypic separation associated with genotypic variation that was likely not driven by outlier strains.

Although we found a significant QTL associated with the elevation of isolation location, we did not recapitulate the QTL for latitude observed in the previous study (Andersen *et al.* 2012). This difference is likely because the previous mapping appears to be driven by strains with extreme latitude values, similar to our elevation mapping. The larger strain set in this analysis reduces the effect of these outliers on the GWA mapping, and, thus, the QTL is no longer significant. However, we did observe a QTL associated with latitude just below the Bonferroni-corrected significance threshold on the right arm of chromosome V. This suggestive QTL is in close proximity to the QTL for temperature. It is possible that the moderate association seen with latitude is representative of a real association with temperature, as the two traits are highly correlated. In our dataset, we see a high negative correlation between the absolute value of latitude and temperature (*rho* = −0.827) and between latitude and temperature (*rho* = −0.795). Unfortunately, the latitude and longitude recorded for a particular strain are not always precise, especially for strains with older isolation dates, which were not recorded as accurately. One-tenth of a degree of latitude can distinguish between large cities but could cover up to 11.1 km of distance. The function we used to determine elevation of strain isolation used these imprecise latitude and longitude coordinates, potentially resulting in a range of small to sizeable errors. These estimations could affect not just the geographic traits but the weather traits as well, which are calculated based on the geographic coordinates. However, we do not expect this estimation to have a large effect, as we found weather data between stations up to 150 km apart to be highly correlated (*rho* = 0.707; see *Materials and Methods*).

We chose to assess weather conditions for each of the 149 strains over three time periods: 3 months, one year, and three year. A duration of 3 months was chosen to define the weather contemporary to the date of isolation; one year was chosen to evaluate the average weather patterns a strain must be able to survive in nature; three year was chosen to define the climate of the isolation location. Using data from three year could help us understand the average long-term climate conditions for each strain, by eliminating any unusual weather patterns during the year of isolation that are not representative of the overall average environmental conditions. The relative humidity trait maps to the same chromosome positions for all three time periods assayed. Previous studies have also shown that *C. elegans* tend to be found in humid regions (Frézal and Félix 2015). The same QTL on the right arm of chromosome V was observed for both relative humidity and temperature. Because relative humidity depends on air temperature, these traits are expected to be correlated. In our dataset, we see a high correlation between temperature and relative humidity (*rho* = −0.675). Although the average temperature maps with only the three-year data set, we observed a QTL at the same position for minimum daily temperature and another QTL just below the significance threshold for the average one-year temperature mapping dataset. Additionally, this QTL was mapped for other weather traits such as maximum daily dew point and variance of wind direction (Figure S1). Similar to relative humidity, dew point is dependent on (and highly correlated with) ambient temperature. However, wind direction and temperature are more difficult to relate to one another. Perhaps, this QTL represents residual population structure shared by strains at this location and is not suggestive of underlying mechanisms impacting fitness in differing environmental conditions. Volkers *et al.* (2013) attempted to characterize such genomic and phenotypic diversity in wild *C. elegans* populations and discovered major hotspots of polymorphic genes on the left arm of chromosome II and the right arm of chromosome V. Maybe the higher genetic diversity observed at these positions is controlling the observed QTL for temperature and other weather traits. Regardless, we observe several QTL that provide evidence that one or more variants on chromosome V are associated with differences in long-term average temperature where *C. elegans* strains are isolated from nature.

The QTL for temperature was evaluated further because it could be controlled in a laboratory setting. The competition assay between JU847 and CX11314 showed that CX11314 had a higher selective advantage than JU847 at both high and low temperatures. This result might be observed because the laboratory environment cannot completely recapitulate conditions experienced in the wild. Additionally, it is possible that CX11314 is more fit than JU847 regardless of temperature. To test this QTL more thoroughly and eliminate this possibility, it would be necessary to compete multiple high and low temperature strains. Alternatively, because JU847 and CX11314 differ throughout their genomes in unknown ways that could affect overall fitness, testing near-isogenic lines (NILs), in which only the region surrounding the QTL is different between the two strains, would be a better way to test temperature-dependent fitness effects of this QTL. Regardless, we found that the low temperature strain, JU847, was more competitive with the high temperature strain, CX11314, at the lower temperature. This result provides evidence that one or more genetic variants within the QTL could contribute to a higher fitness at specific temperatures. Furthermore, the duration of this experiment was only six weeks, while the QTL was identified for a time span of three year. At the lower temperature, it is possible we could observe a stronger competitive advantage for JU847 over a longer time period.

Although our analyses were unable to identify a single gene or variant that could underlie potential differences in niche specification, our conclusions suggest that different strains are found in unique niches, and at least some of the environmental differences in niches are related to genetic variation among strains. As we expand our collection of wild *C. elegans* strains, we will be able to better define these weather and climate differences. Additionally, longitudinal collection studies with dense sampling, especially in a location with known high species diversity (such as the Hawaiian Islands), would give us more valuable data about how genetic variation in *C. elegans* is related to environmental conditions. We expect that similar data could be analyzed for other species, and allow for investigation of niche specification, specifically in plant species where dense sampling and whole-genome datasets are available.

## Supplementary Material

Supplemental material is available online at www.g3journal.org/lookup/suppl/doi:10.1534/g3.116.035162/-/DC1.
