# Supplementary material for: Correlations of Genotype with Climate Parameters Suggest Caenorhabditis elegans Niche Adaptations
Source: G3 (Bethesda). 2016 Nov 17;7(1):289–98. doi: 10.1534/g3.116.035162 (PMC5217117; doi:10.1534/g3.116.035162)
Supplement: Supplementary file 1 [file 289FigureS1.pdf]

Figure S1. Genome-wide association of all significant geographic, weather, and climate traits. Genome-wide association of various geographic and weather traits are visualized as Manhattan plots (A), phenotypic split by genotype (B), and linkage-disequilibrium plots (C). A) Genomic position is plotted on the x-axis against the negative log-transformed *p*-value on the y-axis. SNVs that are above the Bonferroni-corrected significance threshold, indicated by the dotted grey line, are shown in red and SNVs below the Bonferroni threshold are shown in black. Confidence intervals are represented by the pink bars. B) Box plots show range of phenotypic values separated by genotype at the peak marker locus. The reference genotype (REF) refers to isotypes that share the genotype of the reference isotype, N2. The alternative genotype (ALT) refers to all other isotypes. C) Heat map displays the linkage disequilibrium (LD) between each QTL present in the mappings. Red is in high LD, white is low. QTL with an LD of less than 0.5 are presumed to be distinct QTL.

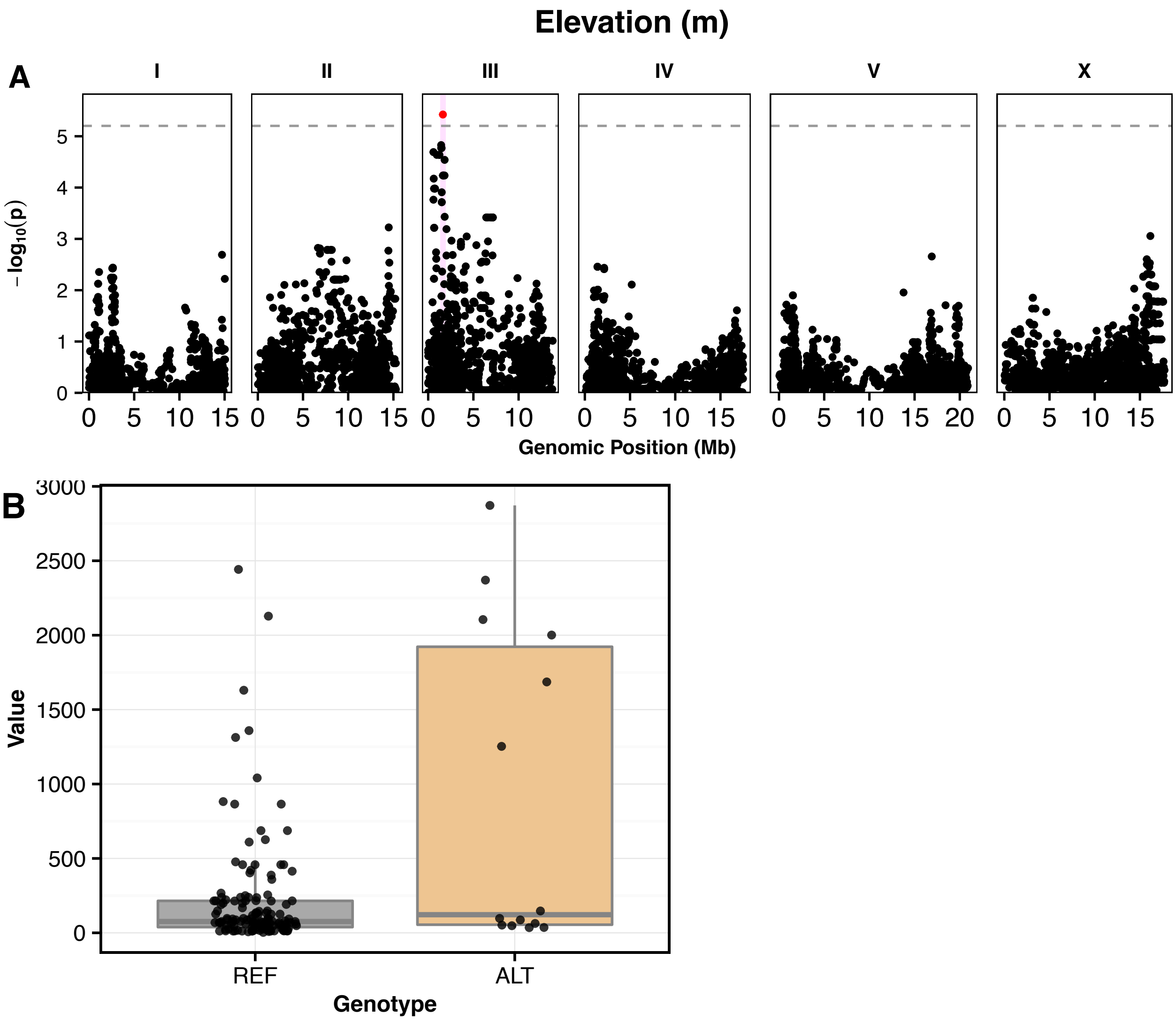

Elevation (m)
A
I
II
III
IV
V
X
−log10(p)
Genomic Position (Mb)
B
Value
Genotype
REF
ALT

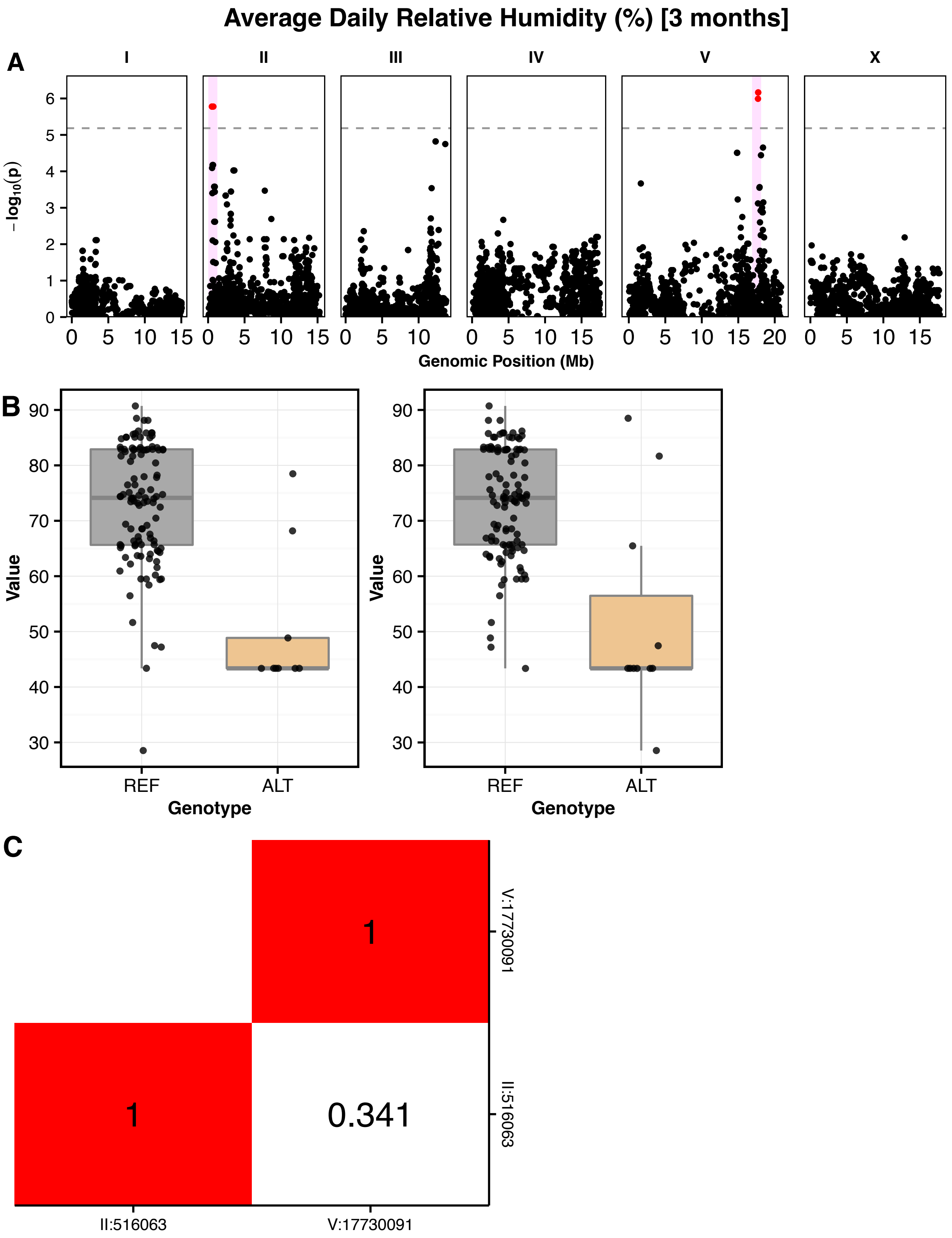
Average Daily Relative Humidity (%) [3 months]
A
I
II
III
IV
V
X
$-\log_{10}(p)$
Genomic Position (Mb)
B
Value
REF
ALT
Genotype
C
1
1
0.341
II:516063
V:17730091

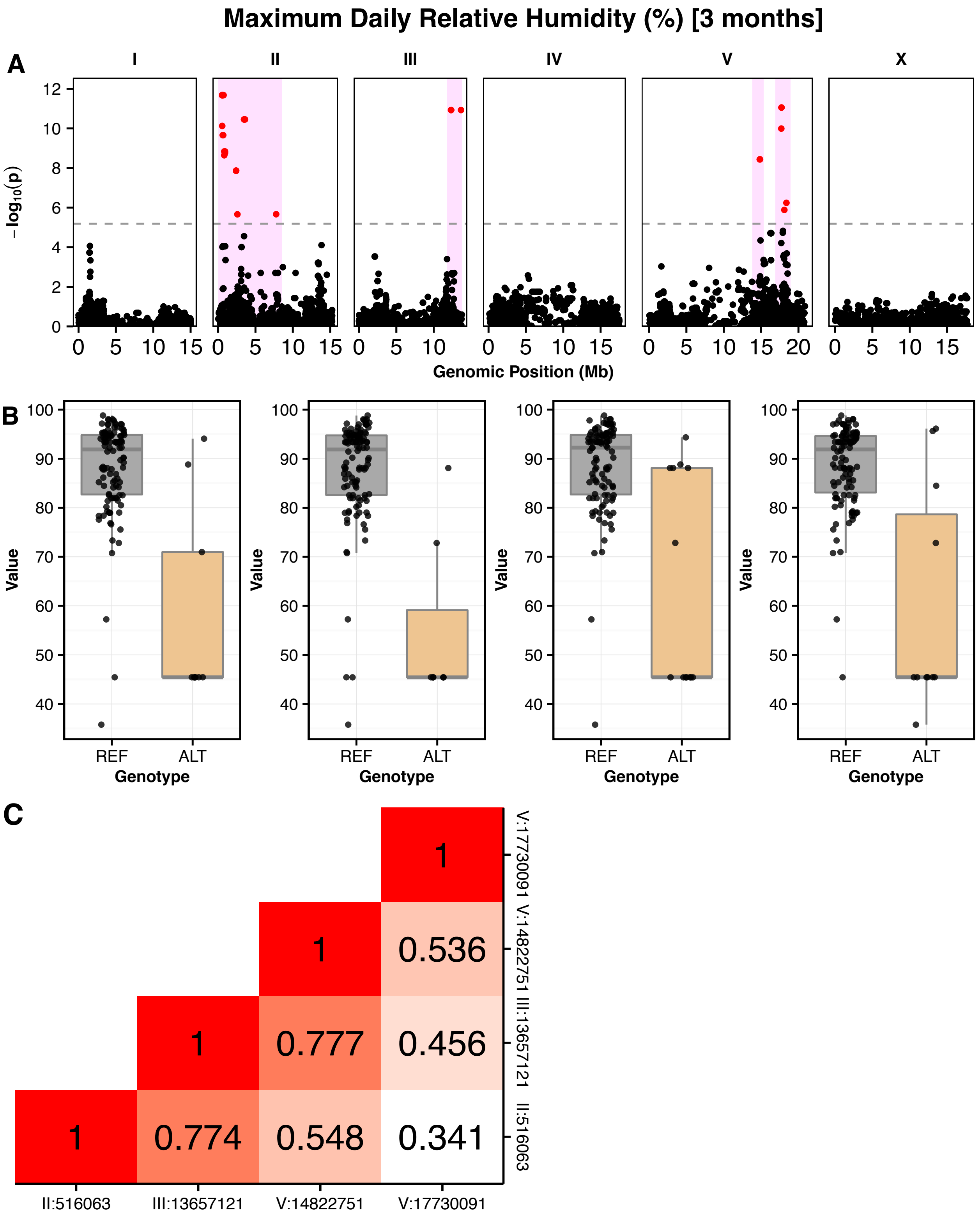

Maximum Daily Relative Humidity (%) [3 months]
A
I
II
III
IV
V
X
−log10(p)
Genomic Position (Mb)
B
Value
REF
ALT
Genotype
C
1
0.536
0.777
0.456
0.774
0.548
0.341
II:516063
III:13657121
V:14822751
V:17730091

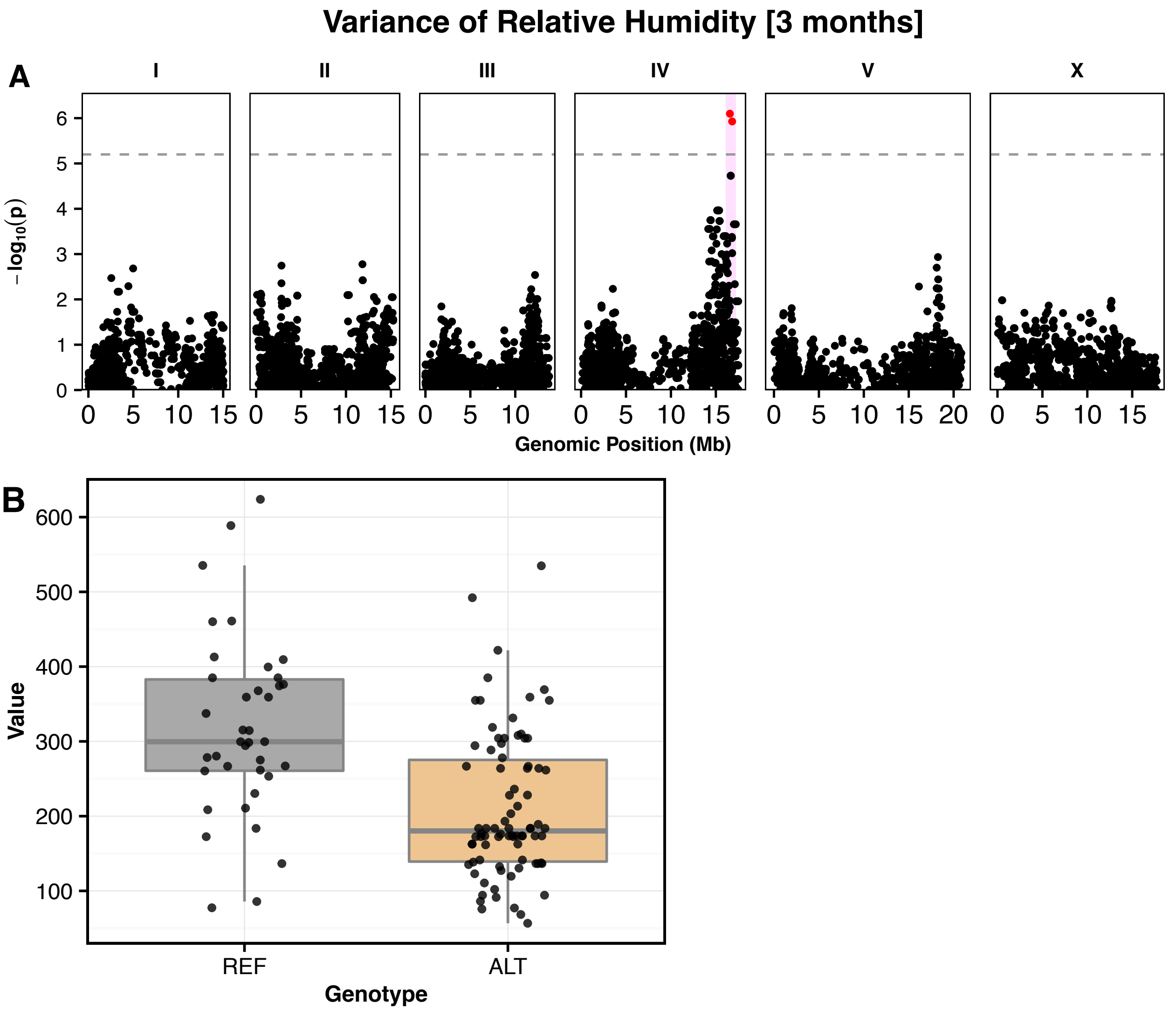
Variance of Relative Humidity [3 months]
A
I
II
III
IV
V
X
−log10(p)
Genomic Position (Mb)
B
Value
REF
ALT
Genotype

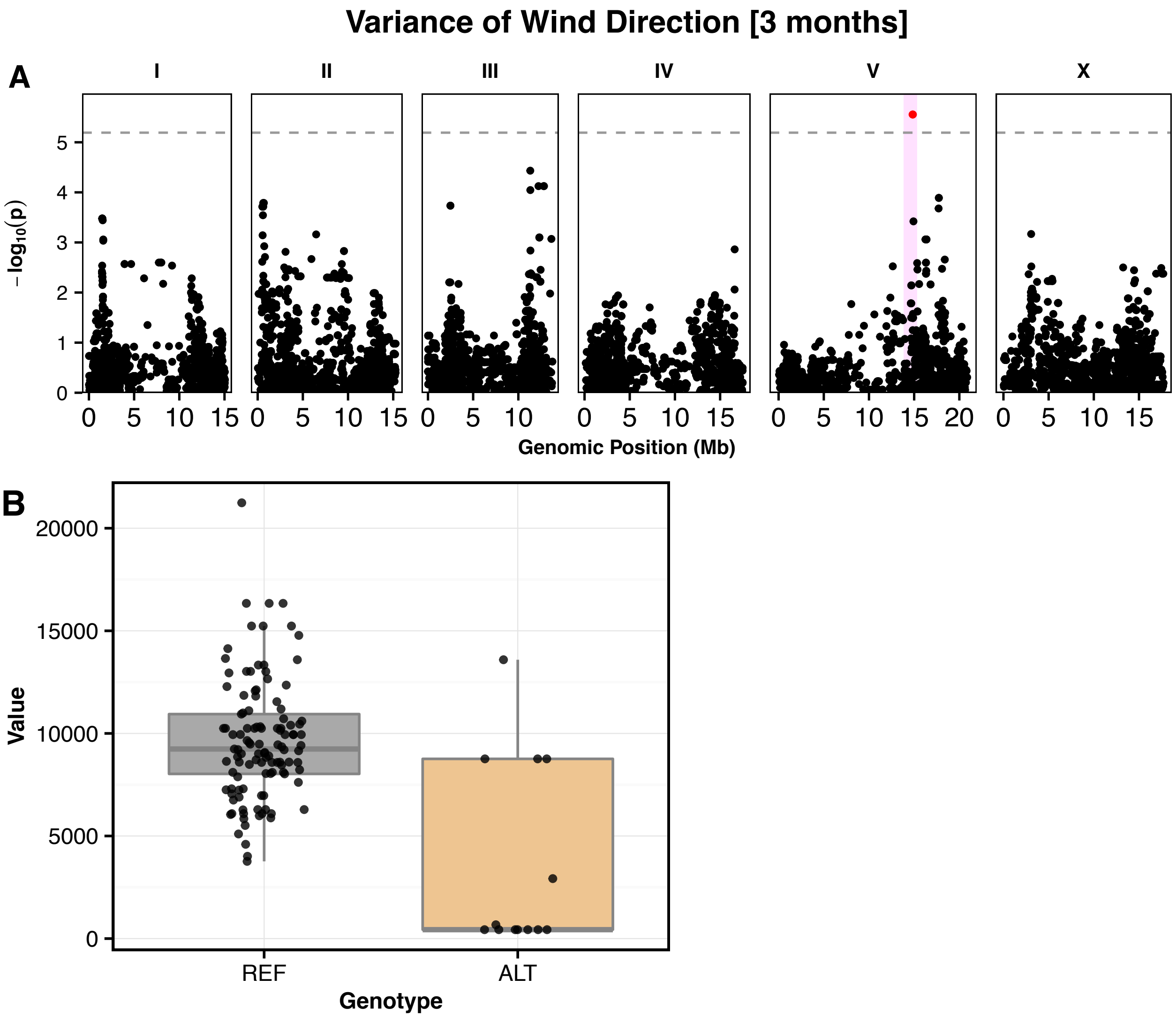
Variance of Wind Direction [3 months]
A
I
II
III
IV
V
X
−log10(p)
Genomic Position (Mb)
B
Value
REF
ALT
Genotype

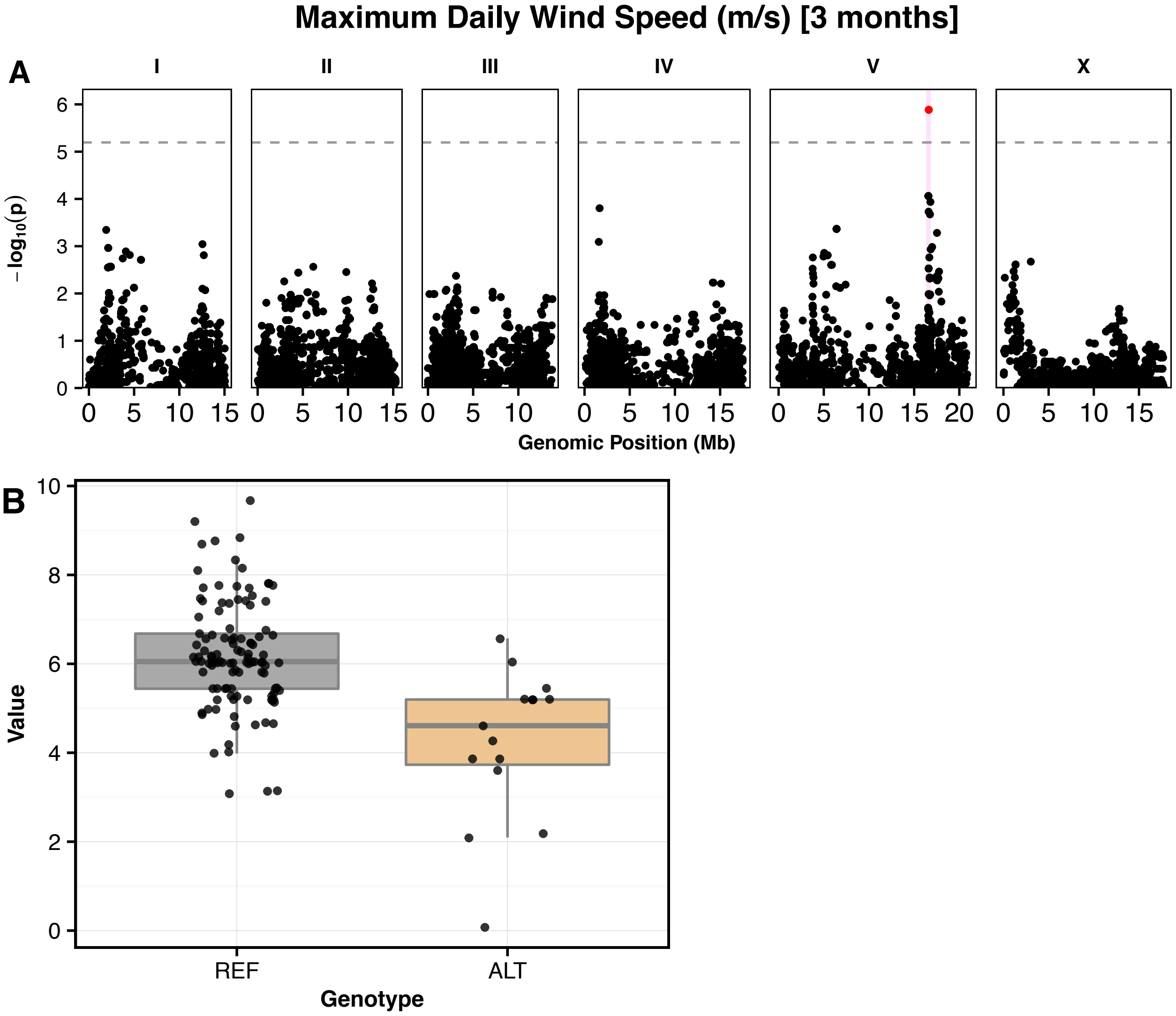

Maximum Daily Wind Speed (m/s) [3 months]
A
I
II
III
IV
V
X
−log10(p)
Genomic Position (Mb)
B
Value
REF
ALT
Genotype

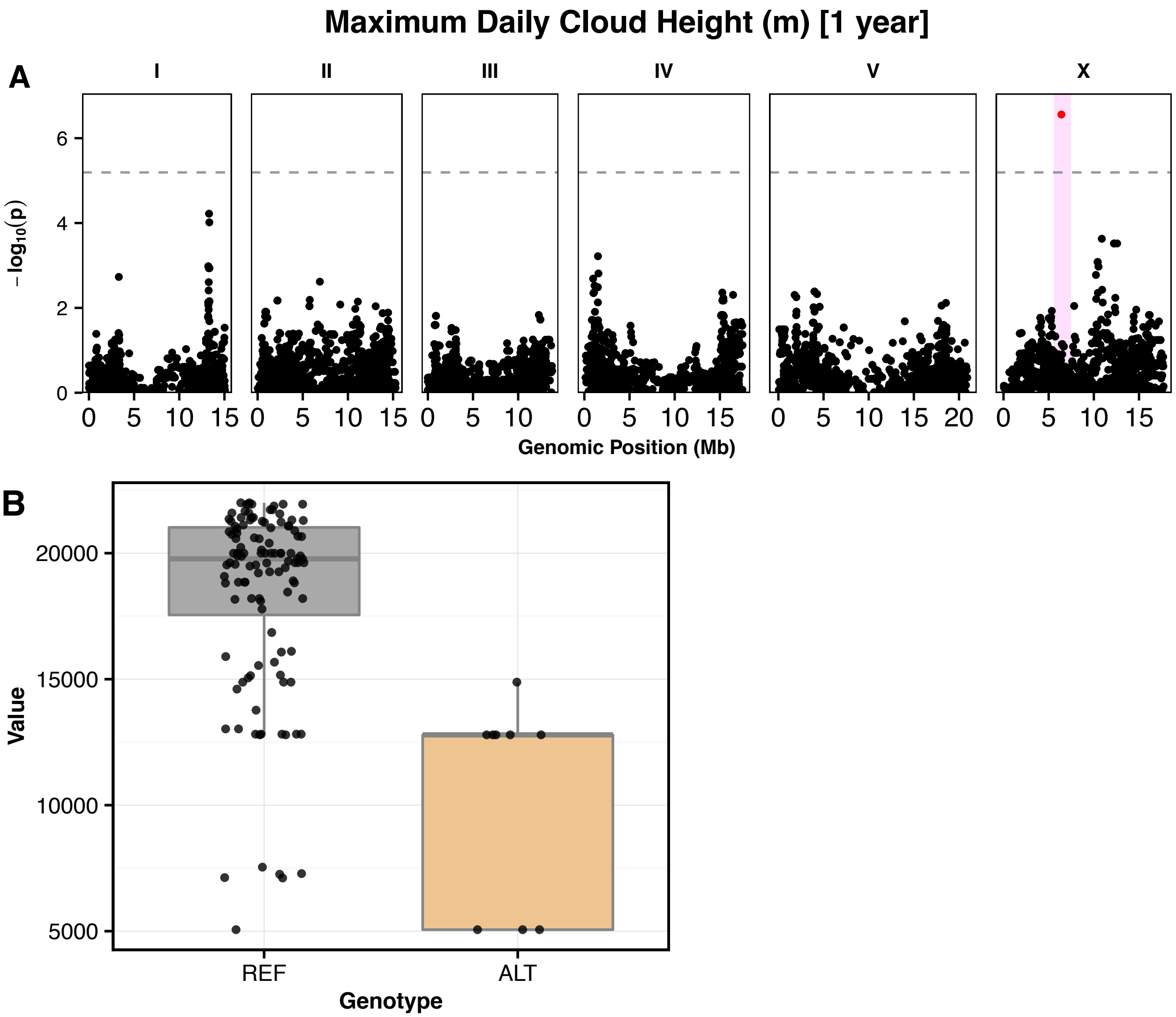
Maximum Daily Cloud Height (m) [1 year]
A
I
II
III
IV
V
X
−log10(p)
Genomic Position (Mb)
B
Value
REF
ALT
Genotype

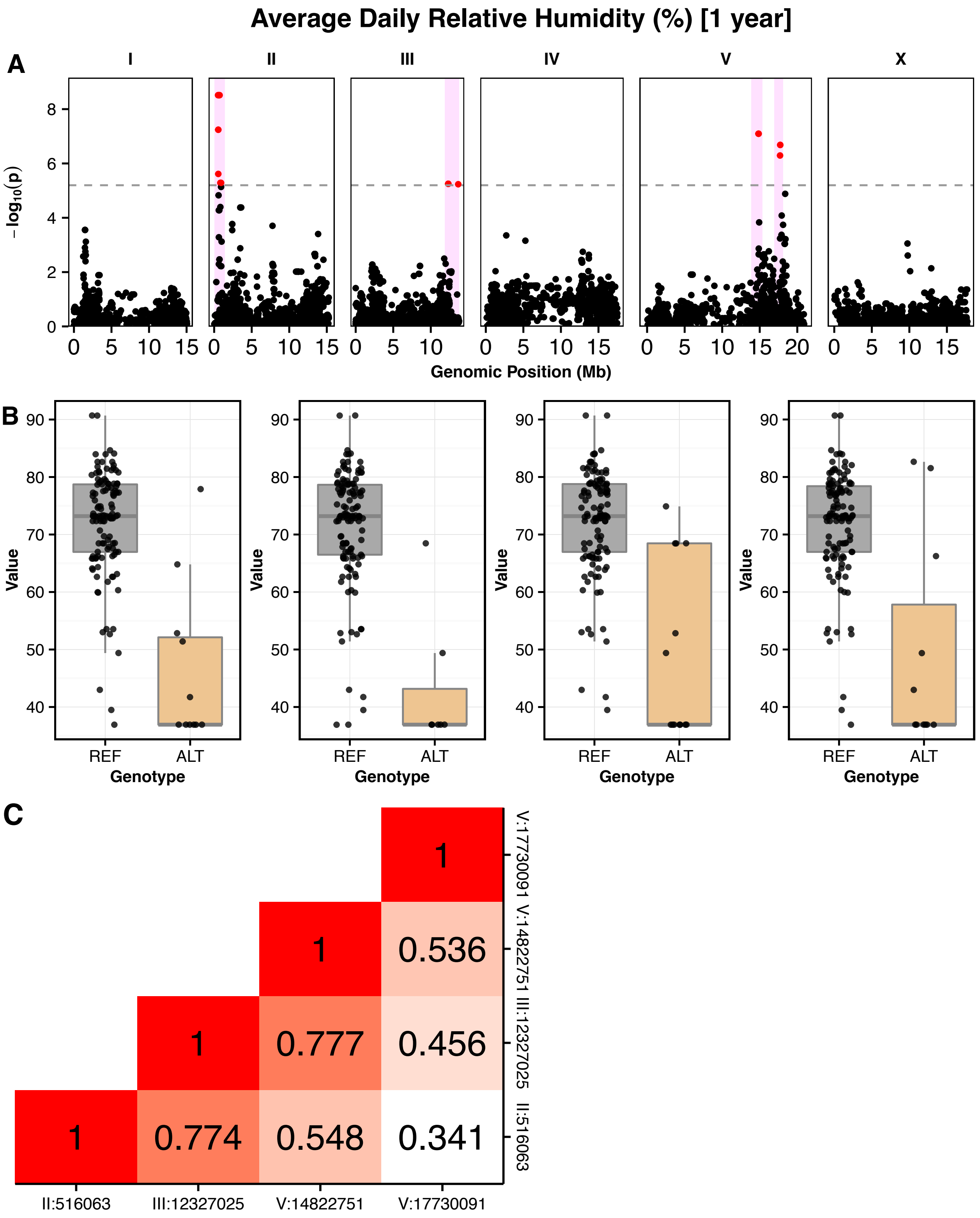

Average Daily Relative Humidity (%) [1 year]
A
I
II
III
IV
V
X
−log10(p)
Genomic Position (Mb)
B
Value
REF
ALT
Genotype
C
II:516063
III:12327025
V:14822751
V:17730091
1
0.774
0.548
0.341
0.777
0.456
0.536

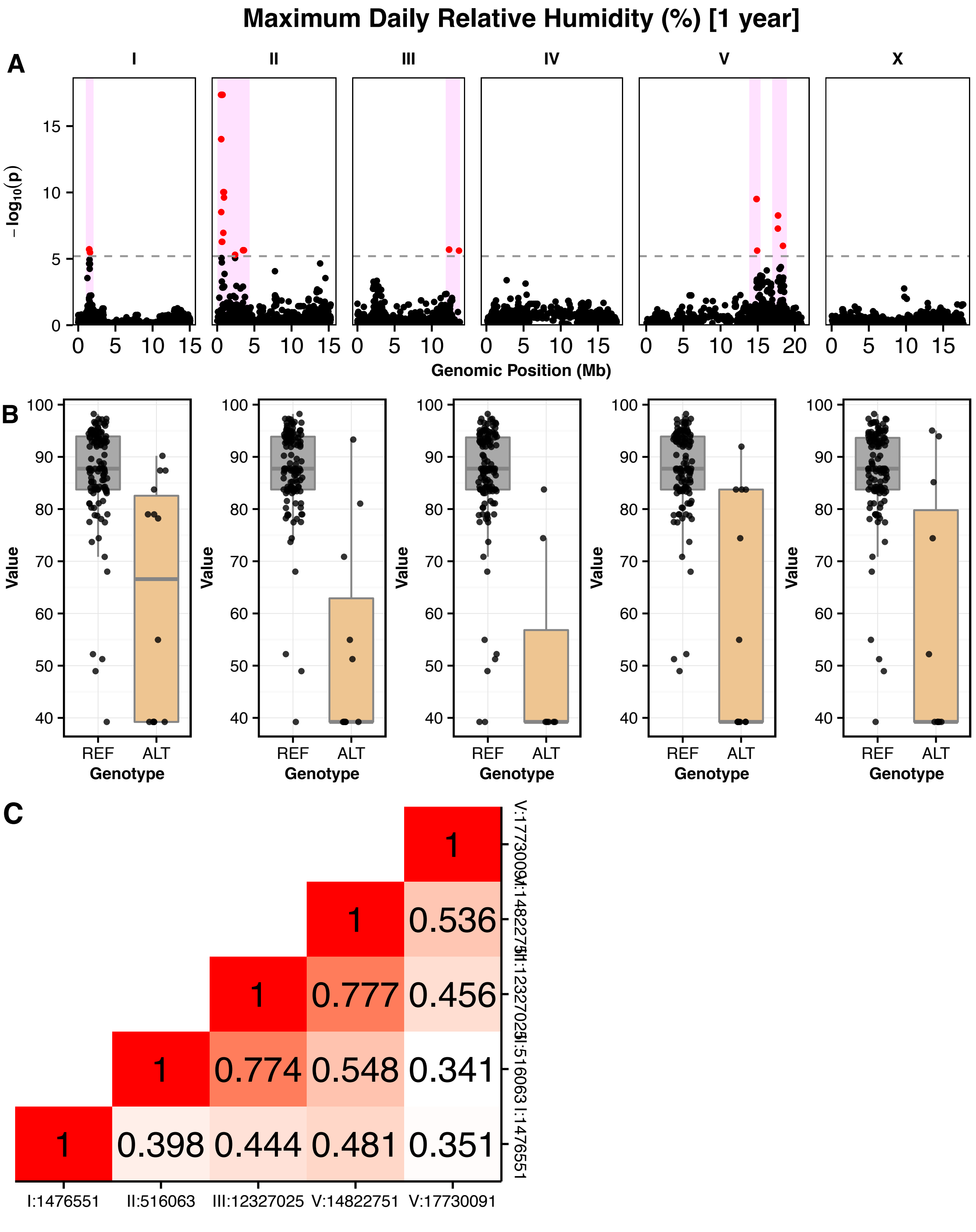

Maximum Daily Relative Humidity (%) [1 year]
A
I
II
III
IV
V
X
−log10(p)
Genomic Position (Mb)
B
Value
REF
ALT
Genotype
C
I:1476551
II:516063
III:12327025
V:14822751
V:17730091

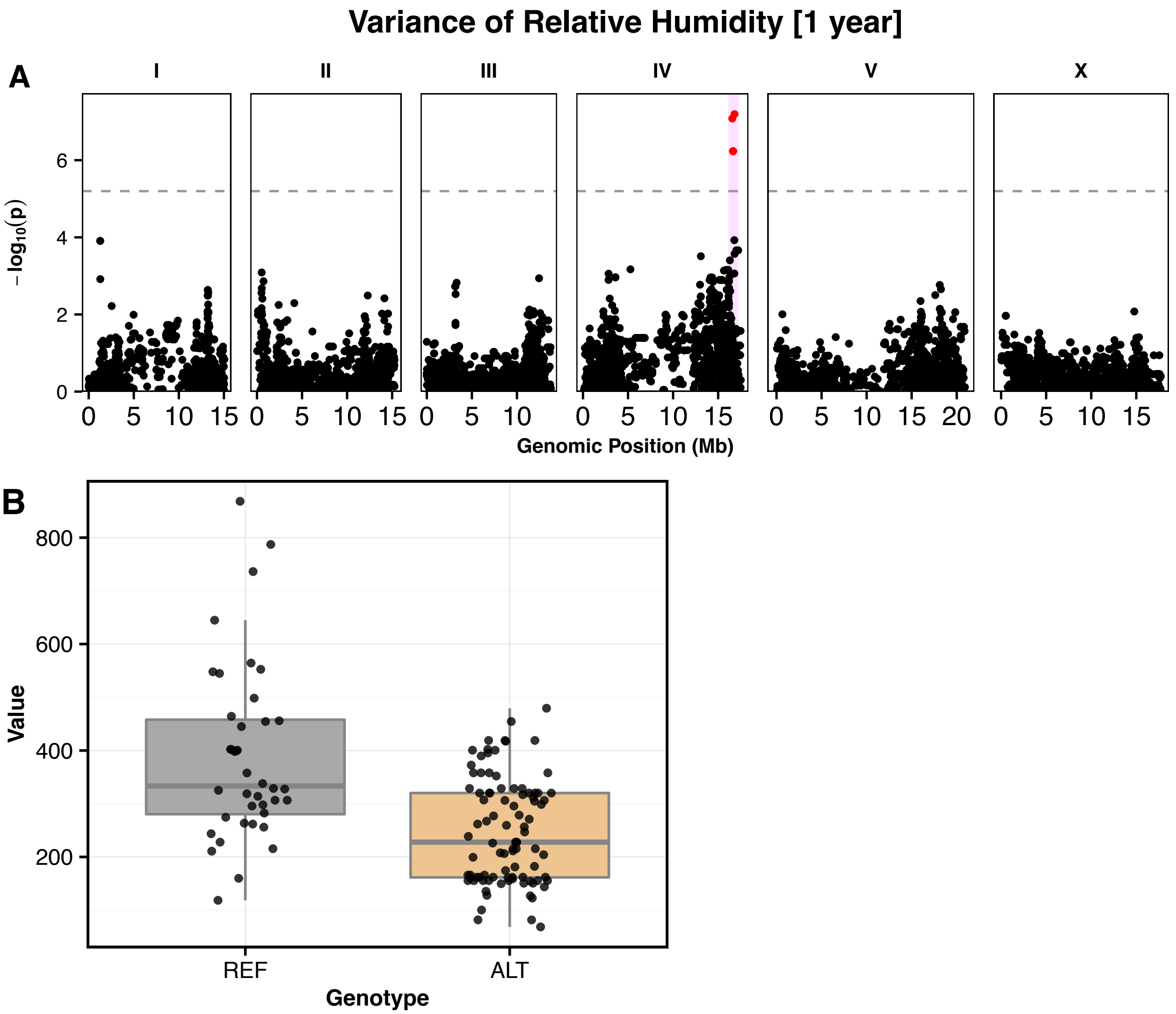

Variance of Relative Humidity [1 year]
A
I
II
III
IV
V
X
−log10(p)
Genomic Position (Mb)
B
Value
REF
ALT
Genotype

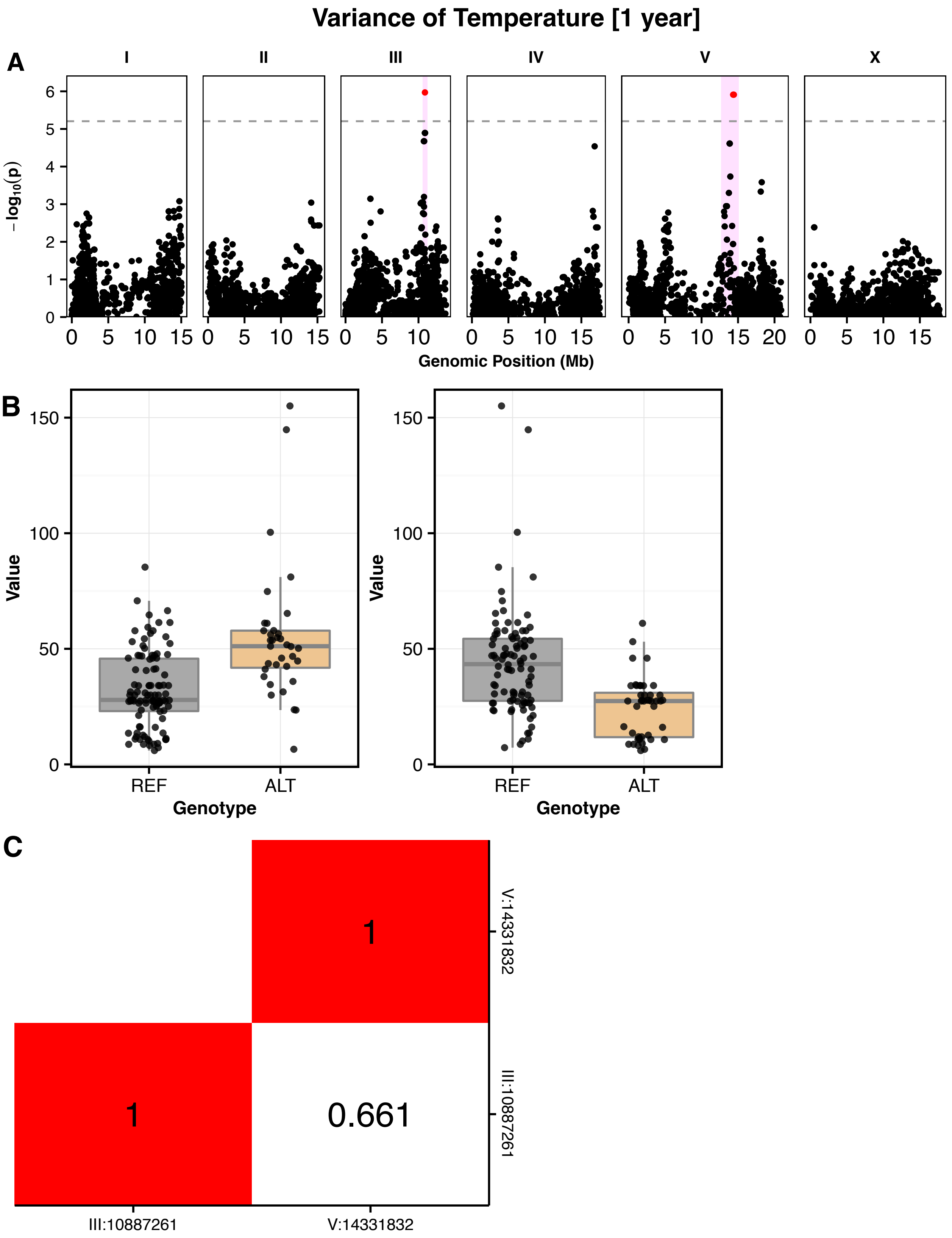

Variance of Temperature [1 year]
A
I
II
III
IV
V
X
−log10(p)
Genomic Position (Mb)
B
Value
REF
ALT
Genotype
C
1
0.661
III:10887261
V:14331832

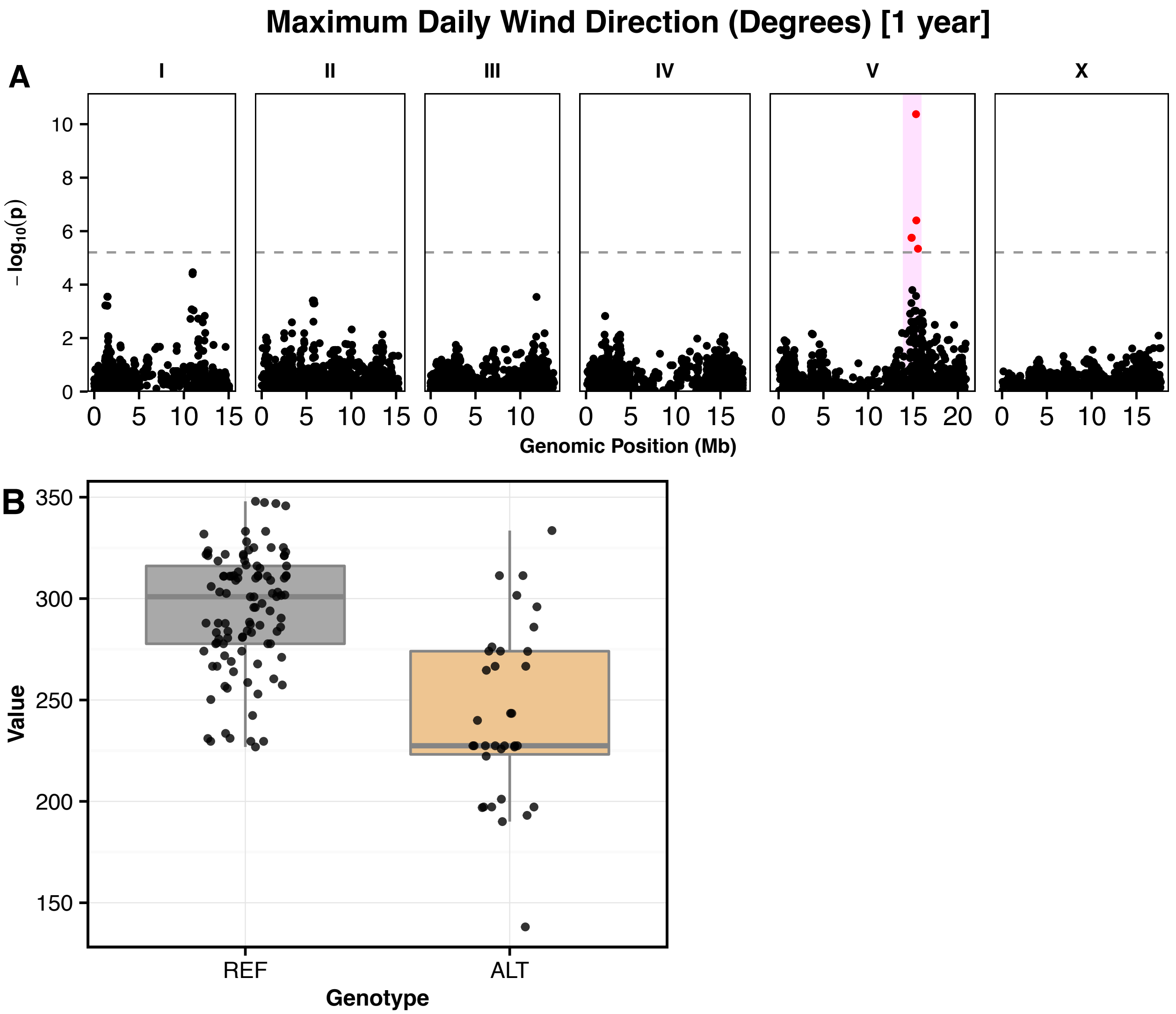

Maximum Daily Wind Direction (Degrees) [1 year]
A
I
II
III
IV
V
X
$-\log_{10}(p)$
Genomic Position (Mb)
B
Value
REF
ALT
Genotype

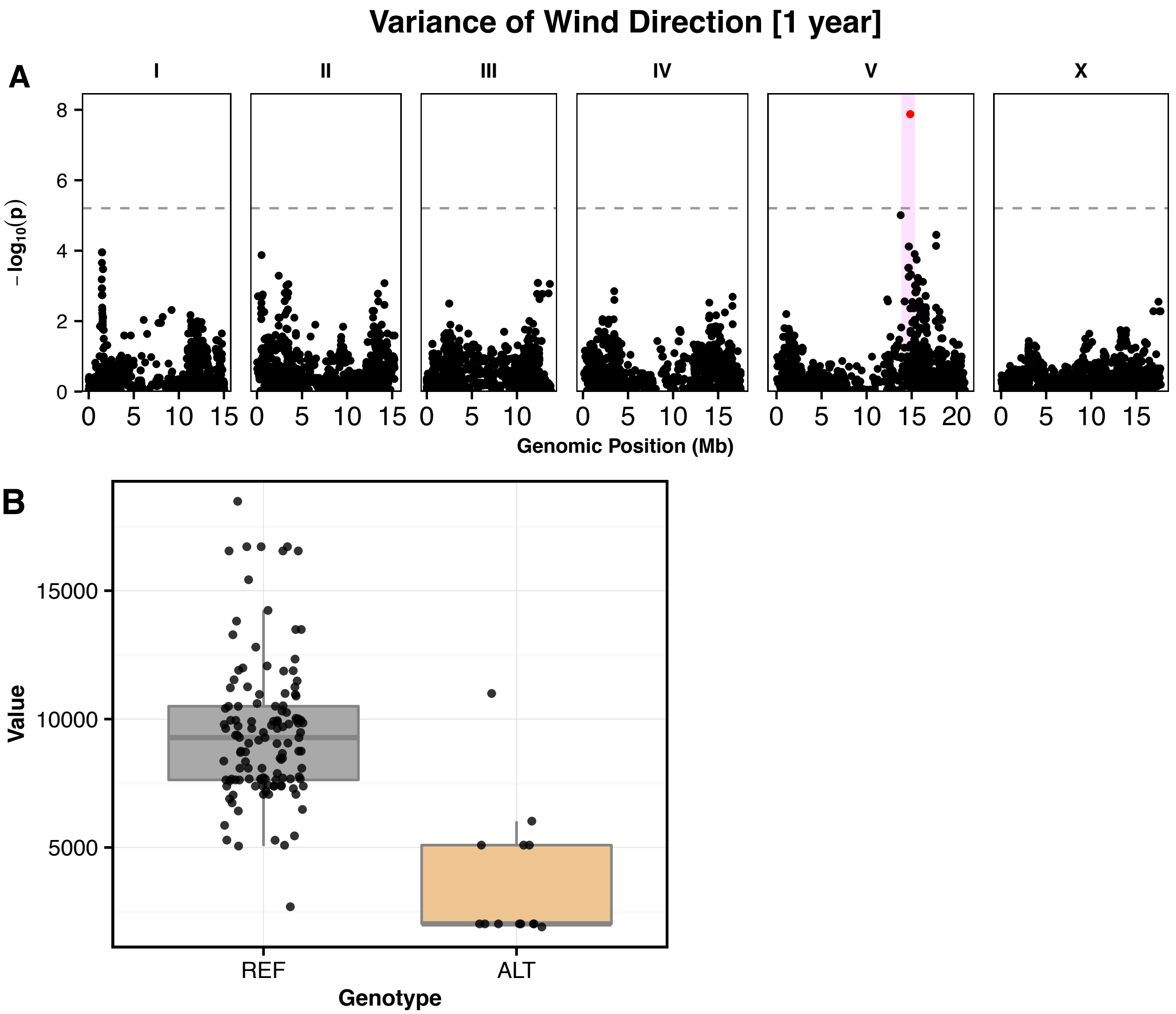

Variance of Wind Direction [1 year]
A
I
II
III
IV
V
X
$-\log_{10}(p)$
Genomic Position (Mb)
B
Value
REF
ALT
Genotype

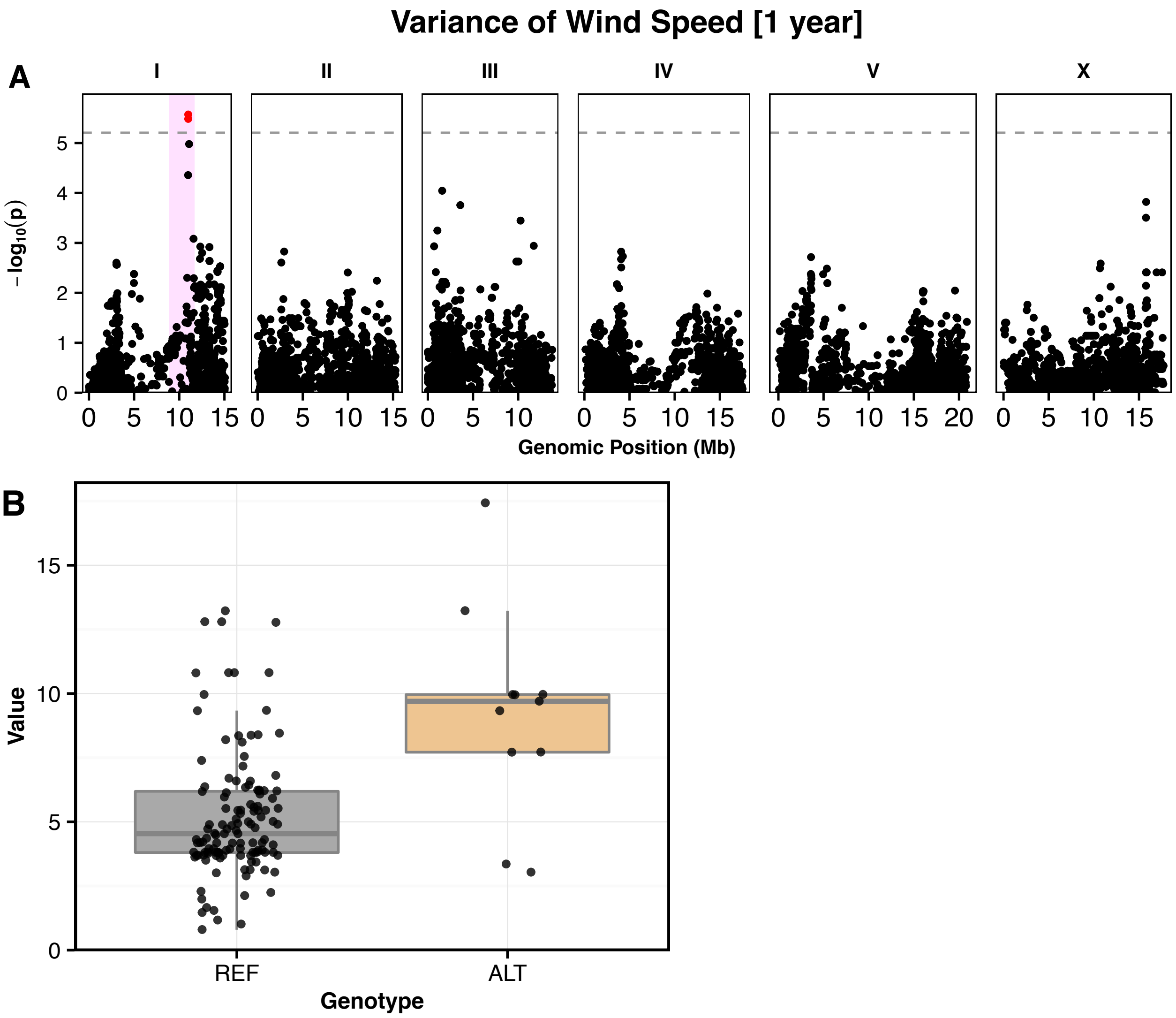

Variance of Wind Speed [1 year]
A
I
II
III
IV
V
X
−log10(p)
Genomic Position (Mb)
B
Value
REF
ALT
Genotype

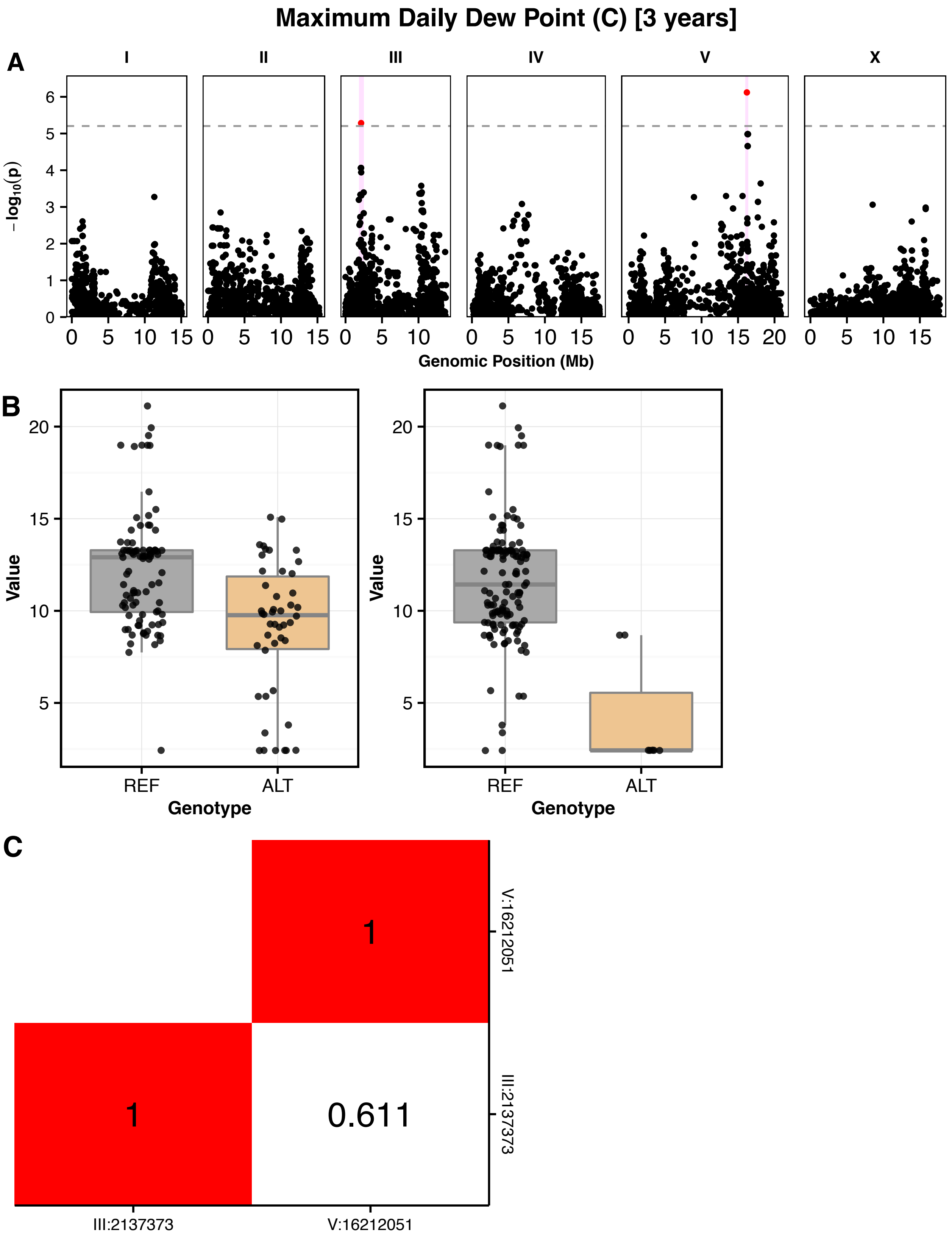
Maximum Daily Dew Point (C) [3 years]
A
I
II
III
IV
V
X
−log10(p)
Genomic Position (Mb)
B
Value
REF
ALT
Genotype
C
V:16212051
III:2137373
1
1
0.611

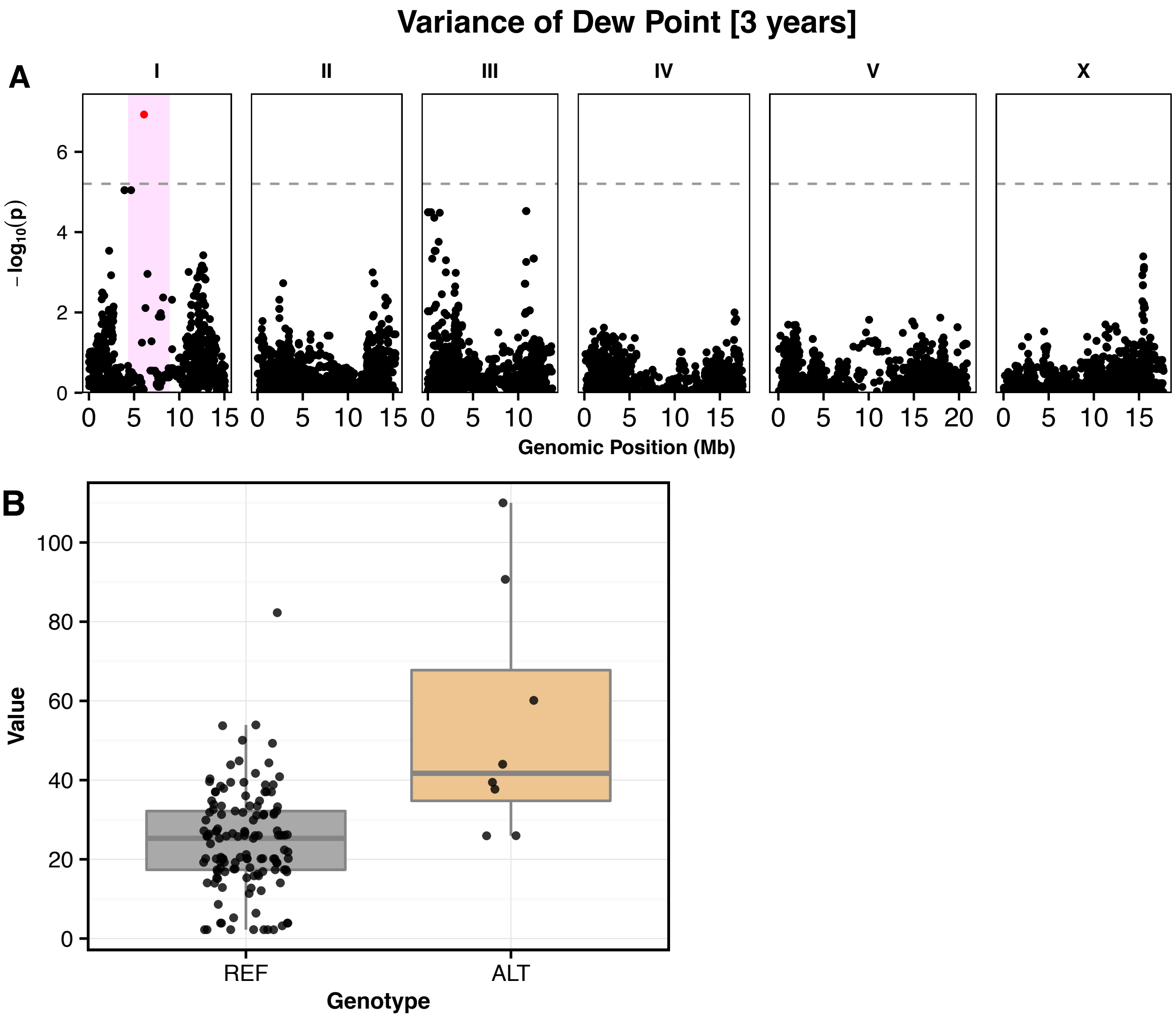
Variance of Dew Point [3 years]
A
I
II
III
IV
V
X
−log10(p)
0
2
4
6
0
5
10
15
20
Genomic Position (Mb)
B
Value
0
20
40
60
80
100
REF
ALT
Genotype

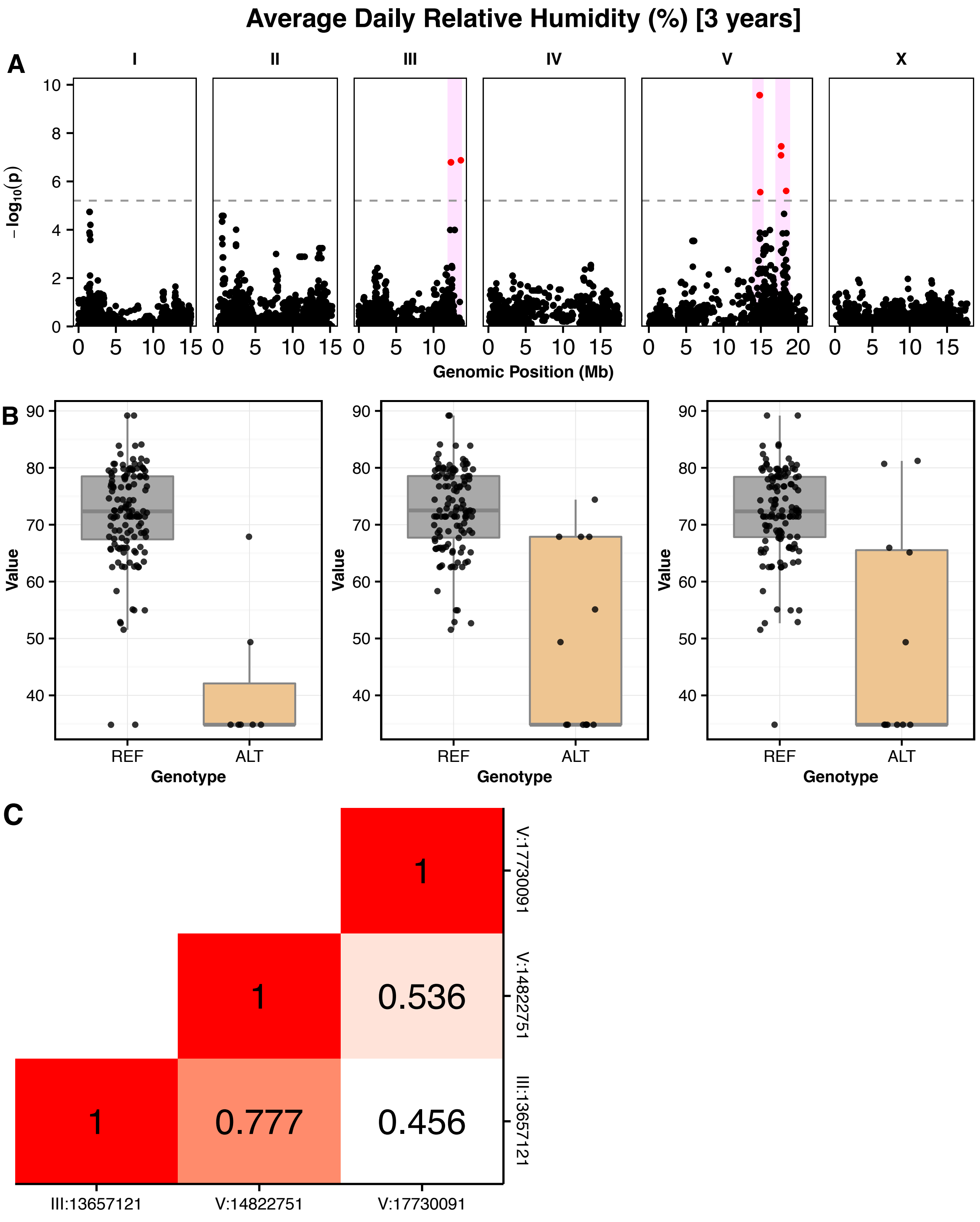

Average Daily Relative Humidity (%) [3 years]
A
I
II
III
IV
V
X
$-\log_{10}(p)$
Genomic Position (Mb)
B
Value
REF
ALT
Genotype
C
III:13657121
V:14822751
V:17730091
1
0.777
0.456
1
0.536
1

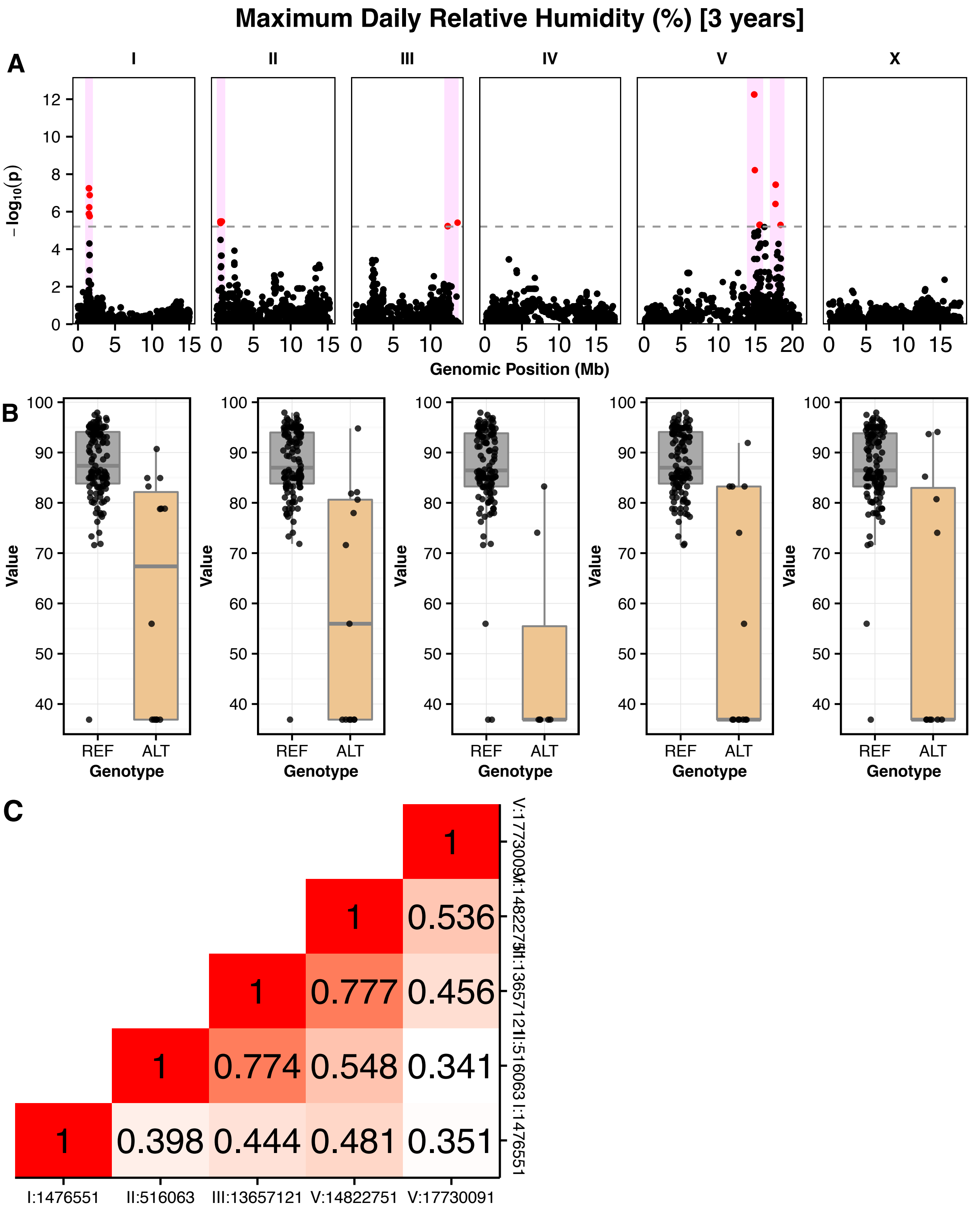

Maximum Daily Relative Humidity (%) [3 years]
A
I
II
III
IV
V
X
−log10(p)
Genomic Position (Mb)
B
Value
REF
ALT
Genotype
C
I:1476551
II:516063
III:13657121
V:14822751
V:17730091
1
0.398
0.444
0.481
0.351
0.774
0.548
0.341
0.777
0.456
0.536

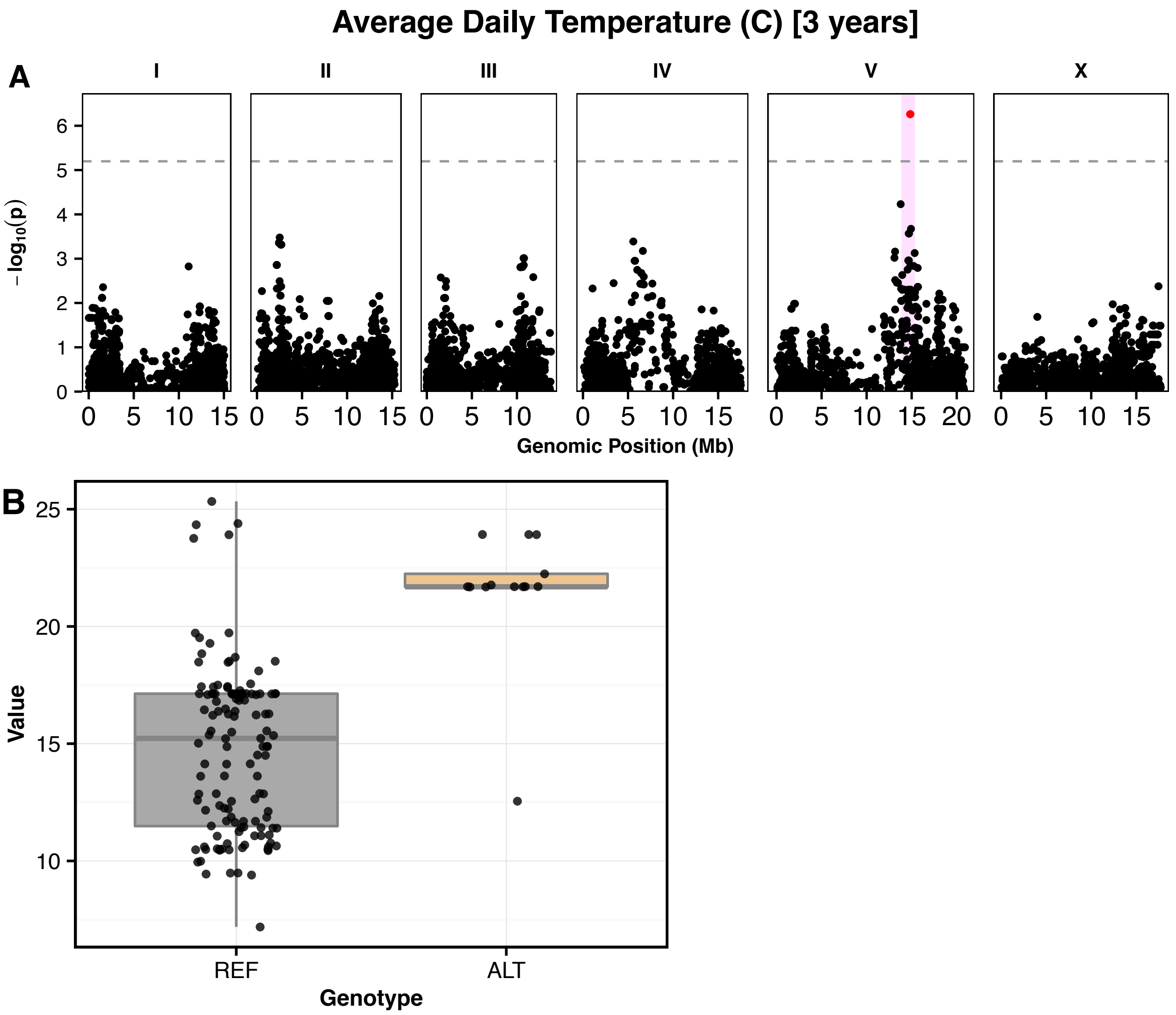

Average Daily Temperature (C) [3 years]
A
I
II
III
IV
V
X
$-\log_{10}(p)$
Genomic Position (Mb)
B
Value
REF
ALT
Genotype

Minimum Daily Temperature (C) [3 years]

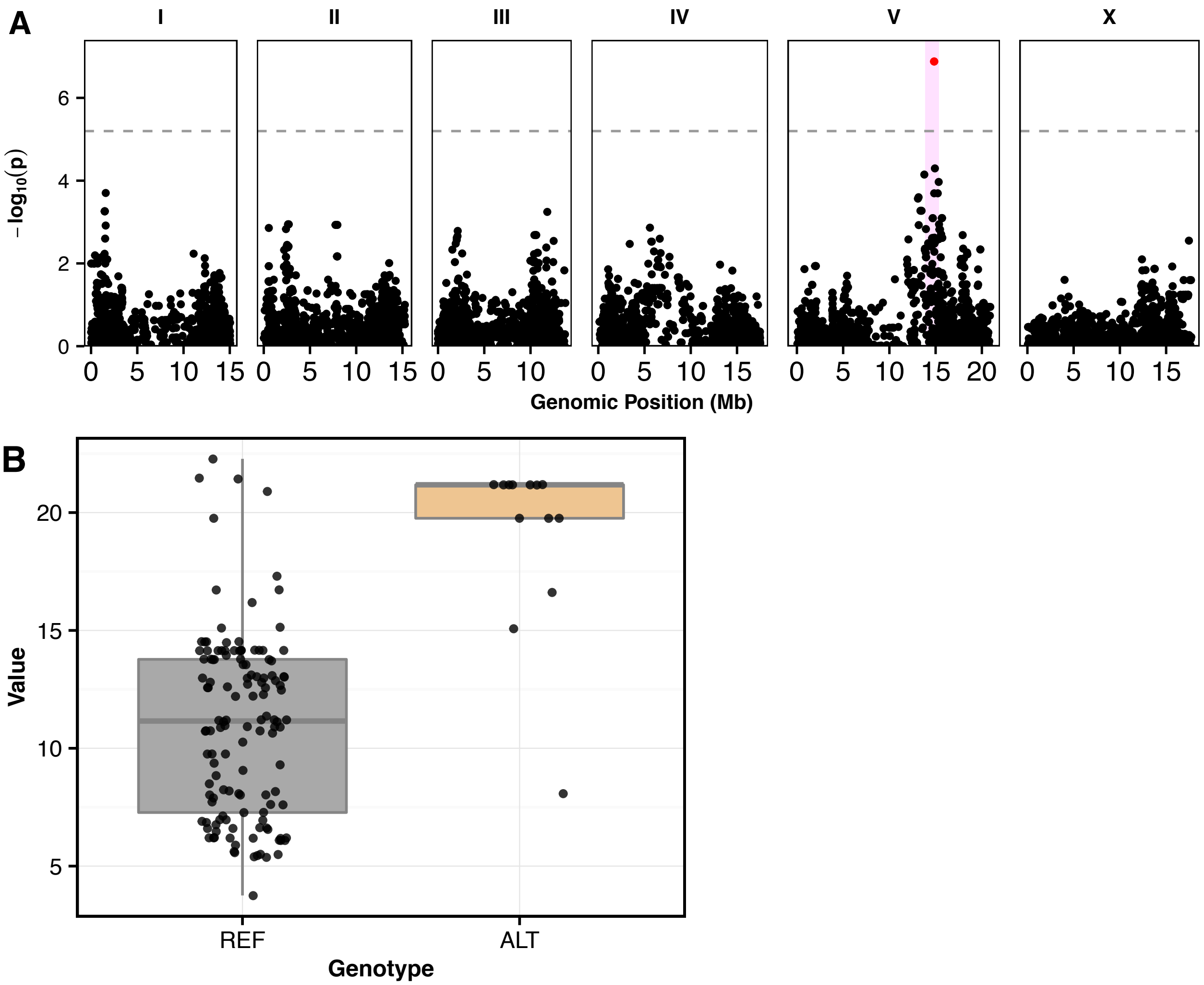

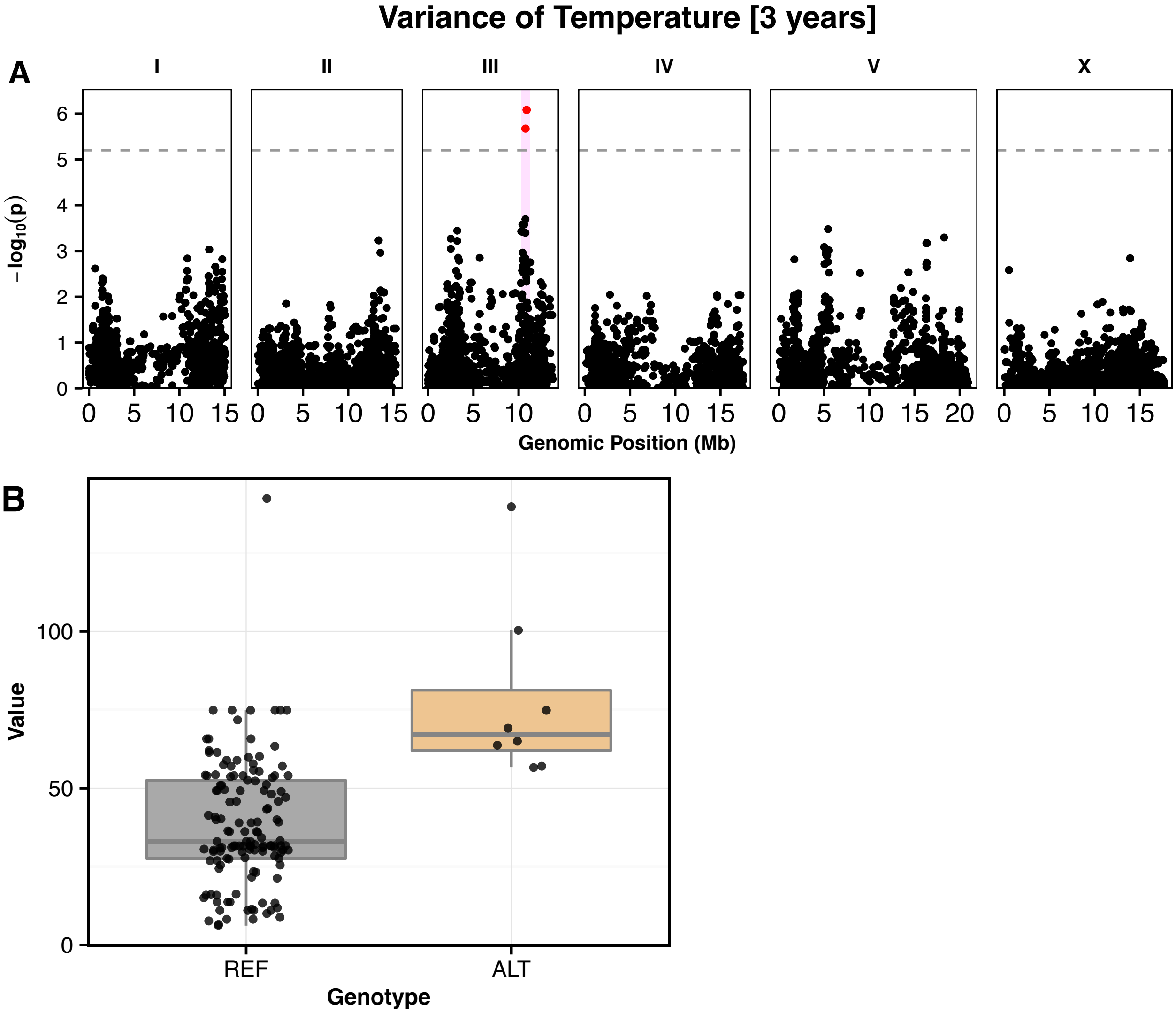

Variance of Temperature [3 years]
A
I
II
III
IV
V
X
−log10(p)
Genomic Position (Mb)
B
Value
REF
ALT
Genotype

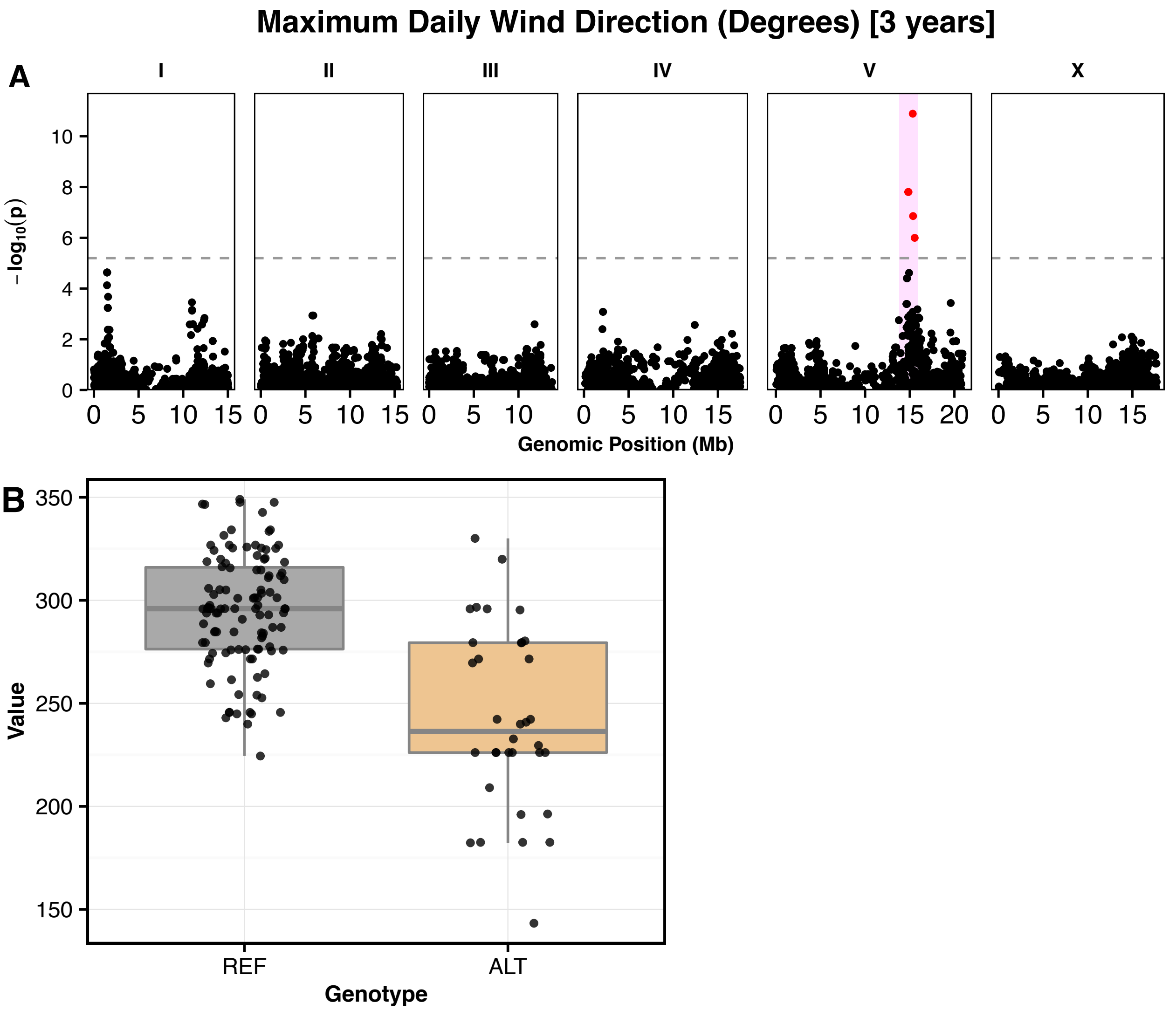

Maximum Daily Wind Direction (Degrees) [3 years]
A
I
II
III
IV
V
X
−log10(p)
Genomic Position (Mb)
B
Value
Genotype
REF
ALT

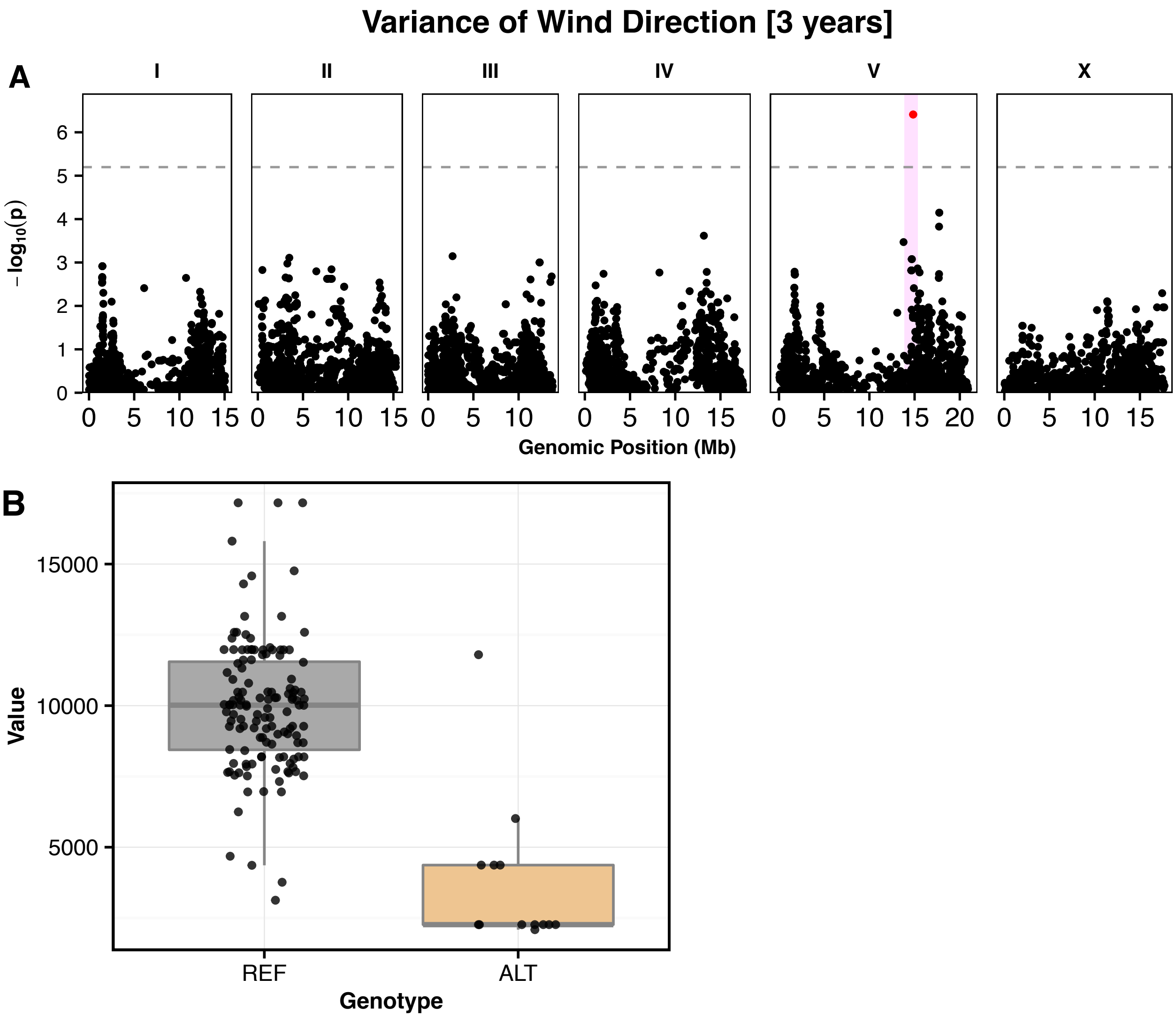

Variance of Wind Direction [3 years]
A
I
II
III
IV
V
X
$-\log_{10}(p)$
Genomic Position (Mb)
B
Value
REF
ALT
Genotype

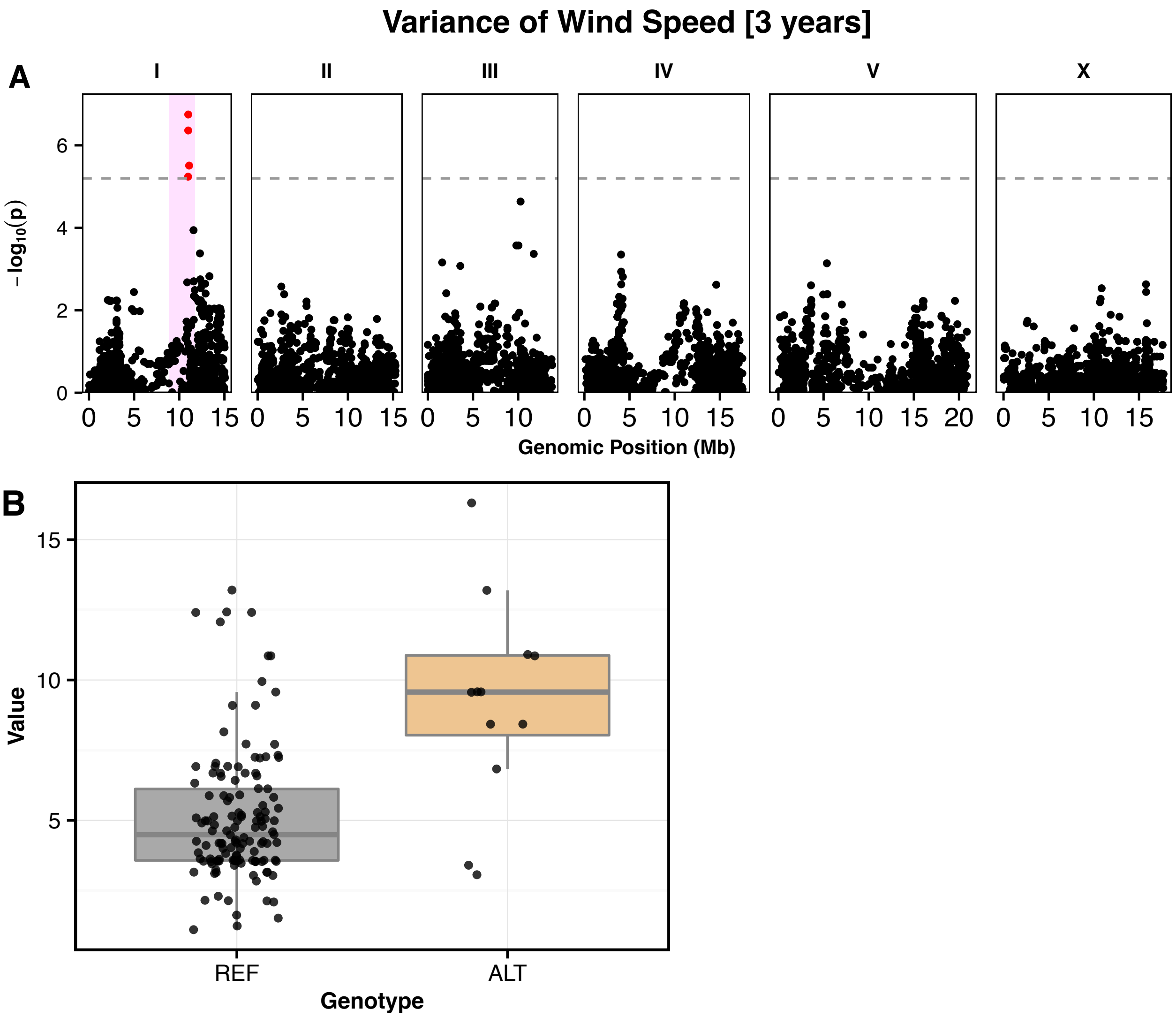
Variance of Wind Speed [3 years]
A
B
I
II
III
IV
V
X
−log10(p)
Genomic Position (Mb)
Value
Genotype
REF
ALT
