# Supplementary material for: Correlations of Genotype with Climate Parameters Suggest Caenorhabditis elegans Niche Adaptations
Source: G3 (Bethesda). 2016 Nov 17;7(1):289–98. doi: 10.1534/g3.116.035162 (PMC5217117; doi:10.1534/g3.116.035162)
Supplement: Supplementary file 2 [file 289FileS1.docx]

File S2: Genome-wide marker association by trait and QTL significance. (.csv, 14 MB)

Available for download as a .csv file at:

http://www.g3journal.org/lookup/suppl/doi:10.1534/g3.116.035162/-/DC1/FileS2.csv
